# Premature senescence is regulated by crosstalk among TFEB, the autophagy lysosomal pathway and ROS derived from damaged mitochondria in NaAsO_2_-exposed auditory cells

**DOI:** 10.1038/s41420-024-02139-4

**Published:** 2024-08-28

**Authors:** Yuna Suzuki, Ken Hayashi, Fumiyuki Goto, Yasuyuki Nomura, Chisato Fujimoto, Makoto Makishima

**Affiliations:** 1 Division of Biochemistry, Department of Biomedical Sciences, Nihon University School of Medicine, Tokyo, Japan; 2 Department of Otolaryngology, Sakura Koedo Clinic, Saitama, Japan; 3 Department of Otolaryngology-Head and Neck Surgery, Keio University, Tokyo, Japan; 4 Department of Otolaryngology-Head and Neck Surgery, Tokai University, Kanagawa, Japan; 5 Department of Otolaryngology-Head and Neck Surgery, Nihon University, Tokyo, Japan; 6 Department of Otolaryngology and Head and Neck Surgery, Graduate School of Medicine, The University of Tokyo, Tokyo, Japan

**Keywords:** Macroautophagy, Stress signalling

## Abstract

Age-related hearing loss (ARHL) is one of the most prevalent types of sensory decline in a superaging society. Although various studies have focused on the effect of oxidative stress on the inner ear as an inducer of ARHL, there are no effective preventive approaches for ARHL. Recent studies have suggested that oxidative stress-induced DNA damage responses (oxidative DDRs) drive cochlear cell senescence and contribute to accelerated ARHL, and autophagy could function as a defense mechanism against cellular senescence in auditory cells. However, the underlying mechanism remains unclear. Sodium arsenite (NaAsO_2_) is a unique oxidative stress inducer associated with reactive oxygen species (ROS) that causes high-tone hearing loss similar to ARHL. Transcription factor EB (TFEB) functions as a master regulator of the autophagy‒lysosome pathway (ALP), which is a potential target during aging and the pathogenesis of various age-related diseases. Here, we focused on the function of TFEB and the impact of intracellular ROS as a potential target for ARHL treatment in a NaAsO_2_-induced auditory premature senescence model. Our results suggested that short exposure to NaAsO_2_ leads to DNA damage, lysosomal damage and mitochondrial damage in auditory cells, triggering temporary signals for TFEB transport into the nucleus and, as a result, causing insufficient autophagic flux and declines in lysosomal function and biogenesis and mitochondrial quality. Then, intracellular ROS derived from damaged mitochondria play a role as a second messenger to induce premature senescence in auditory cells. These findings suggest that TFEB activation via transport into the nucleus contributes to anti-senescence activity in auditory cells and represents a new therapeutic target for ARHL. We have revealed the potential function of TFEB as a master regulator of the induction of oxidative stress-induced premature senescence and the senescence-associated secretion phenotype (SASP) in auditory cells, which regulates ALP and controls mitochondrial quality through ROS production.

## Introduction

Age-related hearing loss (ARHL), known as presbycusis, is one of the most prevalent types of sensory decline and a serious health condition affecting one-third of individuals over 65 years old in modern society, which is termed a superaging society, and accumulating evidence indicates that ARHL is closely linked to the development of cognitive decline, social isolation, and depression [1]. However, the treatment options are limited to medical devices such as hearing aids or cochlear implants, and there are no effective preventive approaches for hearing loss. Although various studies have indicated that oxidative stress plays a key role in the development of ARHL [2, 3], the mechanism remains unclear. Recent studies have highlighted that oxidative stress-induced DNA damage responses (oxidative DDRs) drive cochlear cell senescence and contribute to accelerated ARHL [4, 5]. Cellular senescence is an apoptosis-resistant and nonproliferative state induced by various stressors, including oxidative stress-induced DNA damage; senescence is traditionally characterized by irreversible cell cycle arrest [6] and the secretion of proinflammatory proteins such as inflammatory cytokines or chemokines, termed the senescence-associated secretion phenotype (SASP) [7]. Recent studies have revealed that senescent cells accumulate in various organs and/or tissues, including the inner ear, with increasing age and lead to premature aging of the normal cells around them via the SASP and the induction of chronic inflammation, which accelerates tissue dysfunction and the age-related decline in normal organs or in the context of diseases, including ARHL [4, 8]. Therefore, senescent cells have been described as zombie cells [9]. Interestingly, the depletion of p16 (INK4a)- or p21 (Cip1)-positive senescent cells [10, 11] or the administration of senolytic drugs that specifically induce cell death, targeting only senescent cells [12], causes a delay in age-associated disorders and extends the healthy life span. However, the effect of cellular senescence on ARHL remains incompletely understood.

Importantly, the DDR, an intracellular signaling network essential for sensing and resolving damaged DNA is triggered by oxidative stress and induces autophagy (macroautophagy) [13]; autophagy is an intracellular self-degradation process (a bulk protein-degradation system) that involves the fusion of cargo sequestered in double-layer membrane vesicles (termed autophagosomes) with lysosomes to form autolysosomes and enable degradation of the encapsulated contents, including cytoplasmic proteins and organelles [14]. Autophagy and cellular senescence are stress responses derived from DNA damage that maintain homeostasis in cells [15]. The DDR-autophagy axis contributes to the regulation of cellular senescence and SASP [16, 17]. Interestingly, whether autophagy activates or inhibits cellular senescence depends on the type of cell and the kind of stress [18]. Our previous report indicated that H_2_O_2_-induced premature auditory senescence occurred due to autophagic dysfunction due to 4EBP1 phosphorylation in the mammalian target of rapamycin (mTORC1) pathway [19]. We proposed that autophagy induced by oxidative stress might function as a defense mechanism against cellular senescence in auditory cells.

Sodium arsenite (NaAsO_2_) was used as an oxidative stress inducer associated with reactive oxygen species (ROS) in auditory cells in this study. A previous basic science study showed that subchronic arsenic exposure induced the transformation of human bronchial epithelial cells, accompanied by increased ROS generation and autophagy activation through the mTORC1 signaling pathway [20]. Previous clinical studies reported that arsenic exposure in drinking water induced high-tone hearing loss with an audiogram pattern similar to that of ARHL [21]. Interestingly, this hearing loss occurred in even younger patients (12–29 years old) and young mice [22]. Here, we hypothesized that an in vitro model of premature auditory senescence, which is similar to ARHL, could be developed via arsenic exposure in auditory cells, which generates ROS and activates autophagy through mTORC1.

Additionally, we focused on the function of intracellular reactive oxygen species (ROS) in auditory cells because increased ROS levels due to mitochondrial dysfunction are a hallmark of cellular senescence, including in auditory cells [23, 24]; treatment with oxidoreductase cofactors prevents premature senescence by protecting mitochondrial function in auditory cells [25]. Selective autophagy in mitochondria (mitophagy) could decrease intracellular ROS levels, and the enhancement of selective autophagy under oxidative stress can inhibit cellular senescence [26, 27]. However, the roles of ROS as a cytoplasmic signaling factor that induces cellular senescence and autophagy and is derived from damaged or dysfunctional mitochondria remain controversial in auditory cells.

Recent studies demonstrated that transcription factor EB (TFEB), which functions as a master regulator of the autophagy‒lysosome pathway (ALP) [28, 29], is a potential target during aging and the pathogenesis of various age-related diseases [30, 31]. TFEB was identified as the transcription factor that binds to the promotor motif controlling the expression of lysosomal genes or the coordinated lysosomal expression and regulation network element, which consists of genes involved in ALP; these genes are involved in lysosomal biogenesis, lysosomal exocytosis, endocytosis and membrane repair [32, 33], positively regulating autophagosome formation and autophagosome-lysosome fusion [28]. Autophagy (macroautophagy) is a highly conserved eukaryotic cellular process that degrades intracellular macromolecules in lysosomes [34] and is particularly important in nondividing cells such as auditory hair cells [35] and neurons [32] because it maintains homeostasis and protects hearing. Dysfunctional lysosomal storage leads to autophagic dysfunction because the accumulation of undegraded cargoes in the cytoplasm of cells increases due to the decrease in the basal level of mTOR-dependent lysosomal biogenesis if autophagy is induced only temporarily and linked to aging and age-related diseases [36, 37]. Several lysosomal storage diseases (LSDs), which are inherited metabolic disorders caused by defects in lysosomal proteins or lysosomal-related proteins that lead to lysosomal dysfunction, include hearing loss as a clinical symptom [27]. Damage to the lysosomal membrane results in a decrease in intracellular pH in senescent cells, which affects cell survival [38]. Accumulating evidence indicates that dysregulation of lysosomes is one of the hallmarks of senescence. The enhancement of TFEB functions activates the function of the ALP, promoting the clearance of abnormal protein aggregates, such as amyloid beta or Tau, in a model of neurodegenerative diseases [39] or attenuating the degeneration of spiral ganglion neurons (SGNs) following sensory epithelial cell loss in the cochlea of mice and reducing the level of oxidative stress [40]. However, the role of TFEB-dependent ALP as a potential target to effectively prevent auditory cellular senescence remains incompletely understood. In the present study, we investigated the function of the TFEB-regulated ALP and the impact of intracellular ROS in a NaAsO_2_-induced premature inner ear senescence model with the aim of preventing the progression of ARHL.

## Results

### Short exposure to sodium arsenite (NaAsO_2_) induced a premature senescence phenotype in HEI-OC1 cells

**Fig. 1:**
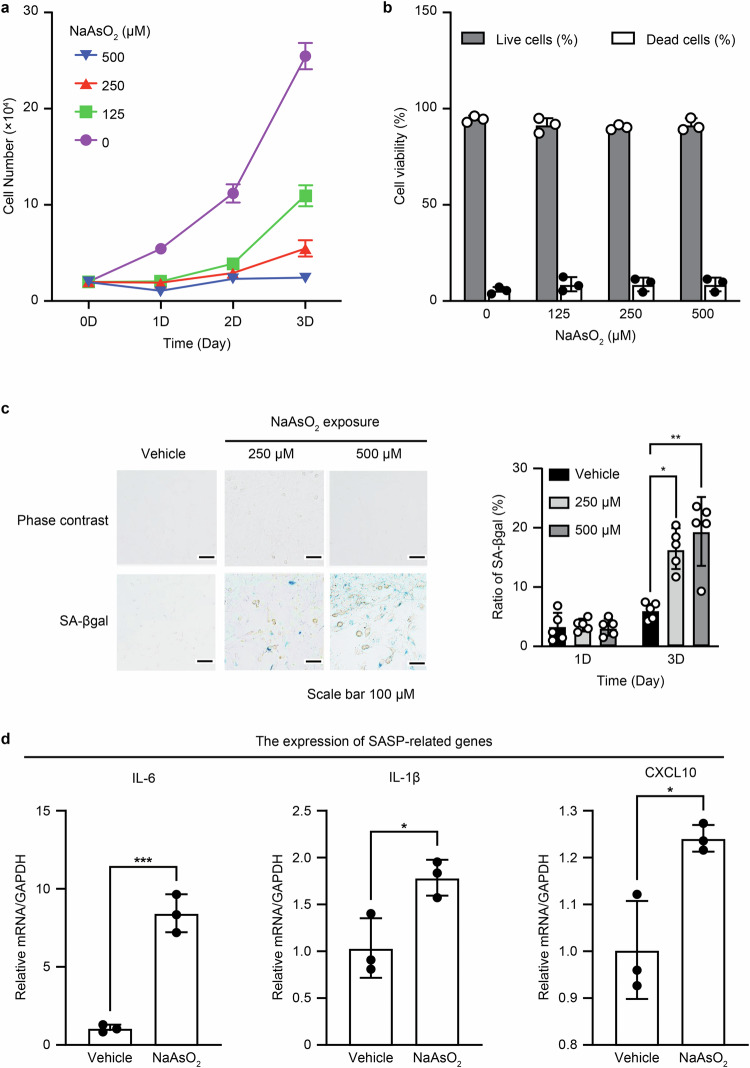
Short exposure to sodium arsenite induces premature senescence in HEI-OC1 cells. Cells were exposed to medium containing different concentrations of NaAsO_2_ (125, 250, and 500 μM) for 1 h. The medium was then replaced, and the cells were incubated in complete medium without NaAsO_2_ for 3 days. **a** Cell proliferation rate; cell numbers were counted every day. NaAsO_2_ concentrations of 125, 250, and 500 μM compared to the control (0 μM). **p < 0.01, Tukey’s test. **b** Cell viability. Cell viability was analyzed by the trypan blue exclusion test 3 days after a short exposure to the indicated concentration of NaAsO_2_. All values are the means ± S.D.s from three or more independent experiments. **c** SA-β-gal-positive cells were quantified by counting more than 200 cells for each sample 3 days after treatment, and the positive rate was calculated from the ratio of positive cells to total cells. All values are the means ± S.D.s from three or more independent experiments. The control condition exhibited no detectable SA-βgal staining. All values are the means ± S.D.s from three or more independent experiments. *p < 0.05, **p < 0.01, Tukey’s test. Scale bar, 100 μm. **d** Relative gene expression of IL-6, IL-1β, and CXCL10 was measured via RT‒qPCR 1 day after a short NaAsO_2_ exposure. All values are the means ± S.D.s from three or more independent experiments. *p < 0.05, ***p < 0.001, ns: not significant, Student’s *t* test.

Senescent cells are in a state of permanent cell cycle arrest and remain viable, unlike apoptotic cells. First, based on prior reports on cellular senescence [19, 24] and concentrations of NaAsO_2_ [22, 41], we assessed the cell proliferation rate and viability after auditory cells were exposed to medium containing different concentrations of NaAsO_2_ (125, 250, and 500 μM) for 1 h and then incubated in complete medium without NaAsO_2_ for 3 days. The cell proliferation rate of cells subjected to a short exposure to NaAsO_2_ decreased in a dose- and time-dependent manner (Fig. 1a), while the cell viability was unchanged, as the ratio of dead cells remained below 3% even 3 days after a short exposure to NaAsO_2_ (Fig. 1b). We regarded this condition as a model of NaAsO_2_-induced premature senescence in auditory cells.

Next, we investigated the phosphorylation of histone H2AX (γH2AX) as a trigger of the DNA damage response (DDR) in auditory cells to confirm whether NaAsO_2_ induces double-strand breaks (DSBs) [42]. γH2AX is an indicator of DSBs and an initiator of the DDR [43]. Immunofluorescence staining analysis indicated that the number of γH2AX foci/cell was significantly higher 24 h after exposure to NaAsO_2_ than that after exposure to vehicle in HEI-OC1 cells (Fig. S2A). This result suggested that the DDR caused by a short NaAsO_2_ exposure might trigger the induction of premature senescence in auditory cells.

We observed the expression of the cyclin-dependent kinases p21 (cdkn1a) and p16 (cdkn2a) to investigate whether NaAsO_2_ exposure induces cell cycle arrest in auditory cells (Fig. S2B; right panel: mRNA level, left panel: protein level; S1A, full-length blots). The expression of p21 was increased and peaked at 12 h, while p16 expression did not change. This result indicated that permanent cell cycle arrest in G1 or G2-M phase occurred in cells subjected to a short exposure to NaAsO_2_ [44]. Finally, we evaluated the positive rate of senescence-associated βgalactosidase (SA-βgal), which is widely used as a cellular senescence marker, after a short exposure to NaAsO_2_ and the expression of senescence-associated secretory phenotype (SASP) genes (IL-6, IL-1β, and cxcl10), which induce senescence in surrounding cells or re-enforce senescent cells themselves as paracrine factors to confirm the induction of the senescence phenotype in auditory cells. SA-βgal-positive cell numbers were significantly increased at 3 days after a short NaAsO_2_ exposure (Fig. 1c), and the cells showed an enlarged, flattened and irregular morphology, which are regarded as characteristics of senescent cells (Fig. S2C, left panel). Positive staining for SA-βgal was mainly observed in these cells. The mean areas of SA-βgal-positive cells were significantly wider than those of SA-βgal-negative cells (Fig. S1C, right panel).

The expression of SASP-related genes (IL-6, IL-1β, and cxcl10) was also significantly elevated in cells subjected to a short NaAsO_2_ (Fig. 1D). These findings indicated that the senescence phenotype was initiated by the DDR due to short exposure to NaAsO_2_ in auditory cells.

### Intracellular ROS derived from damaged mitochondria contribute to NaAsO_2_-induced premature senescence as a second messenger in auditory cells

**Fig. 2:**
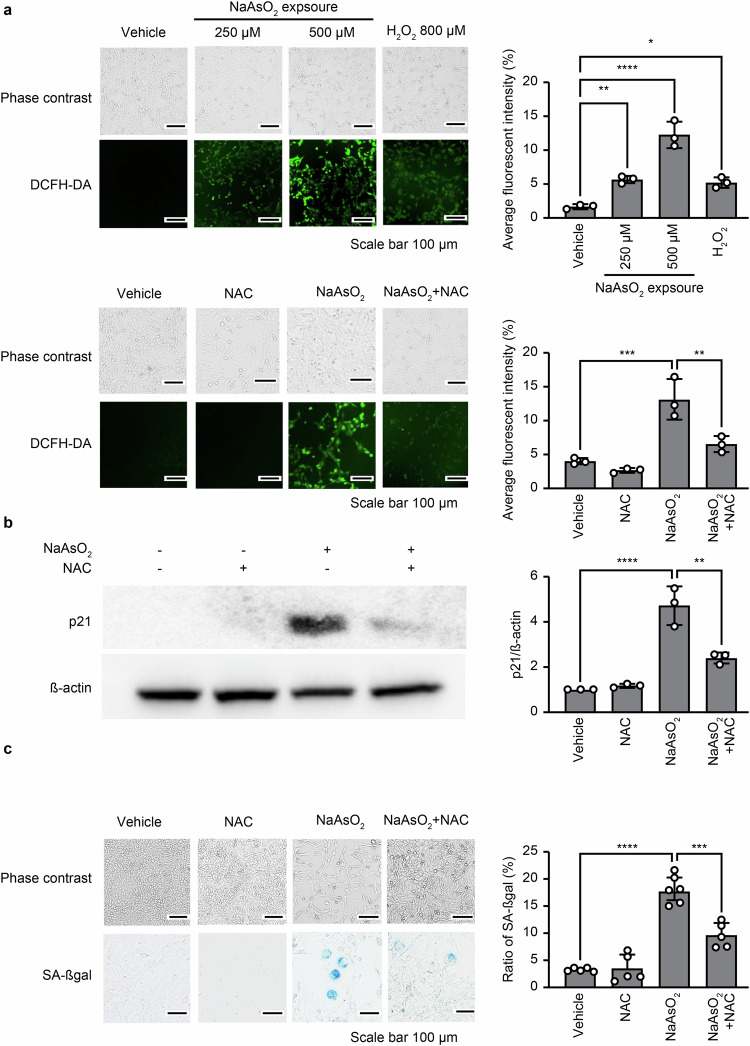
Intracellular ROS produced by damaged mitochondria contribute to NaAsO_2_-induced premature senescence as a second messenger in auditory cells. **a** Upper panel: HEI-OC1 cells treated with NaAsO_2_ (250 and 500 μM for 1 h) or H_2_O_2_ (800 μM for 1 h) were incubated with DCFH-DA (10 μM for 1 h) and then visualized by fluorescence microscopy (KEYENCE) in EBSS, which was added after washing with PBS twice. Lower panel: cells that were pretreated with N-acetylcysteine (NAC) (2 mM for 1 h) and then treated with NaAsO_2_ (500 μM for 1 h) were incubated with DCFH-DA (10 μM for 1 h) and then visualized by fluorescence microscopy (KEYENCE). **b** SA-βgal-positive cells were quantified by counting more than 200 cells for each sample 3 days after the cells were treated with NAC (2 mM for 1 h) and NaAsO_2_ (500 μM for 1 h) as described in (**a**); the positive rate was calculated from the ratio of positive cells to total cells. All values are the means ± S.D.s from three or more independent experiments. The control condition exhibited no detectable SA-βgal staining. All values are the means ± S.D.s from three or more independent experiments. ***p < 0.001, ****p < 0.0001, Tukey’s test. **c** The expression of p21 at the protein level was measured by western blotting at 12 h after treatment with NAC (2 mM for 1 h) and NaAsO_2_ (500 μM for 1 h) as described in (**a**). Western blot analysis was performed as described in the “Materials and methods”. β-Actin was used as a loading control. **p < 0.01, ****p < 0.0001, Tukey’s test. Full-length blots are presented in Supplementary information Fig. S1B.

We evaluated whether exposure to NaAsO_2_ increases intracellular ROS levels derived from damaged mitochondria in HEI-OC1 cells because NaAsO_2_ functions as a second messenger that induces premature senescence in auditory cells, indicating mitochondrial dysfunction [27, 45]. As shown in Fig. 2a upper panel, ROS levels were extremely elevated in a dose-dependent manner at 6 h after a short exposure to NaAsO_2_ and higher than those observed with 800 μM H_2_O_2_, which was used as a positive control; however, this effect was completely suppressed by N-acetylcysteine (NAC), a well-known radical scavenger that has been used to modulate oxidative stress (Fig. 2a lower panel). Importantly, NAC treatment released auditory cells from cell cycle arrest by suppressing the expression of p21 (Fig. 2b; S1B, full-length blots) and led to a decrease in SA-βgal-positive cell numbers in cells with NaAsO_2_-induced premature senescence (Fig. 2c). These results indicate that intracellular ROS derived from damaged mitochondria regulate the cell cycle in auditory cells under oxidative stress, inducing premature senescence due to mitochondrial dysfunction separate from the DDR.

### Short NaAsO_2_ exposure leads to reduced degradation capacity of the autophagy‒lysosome pathway (APL), affecting the induction of premature senescence in HEI-OC1 cells

**Fig. 3:**
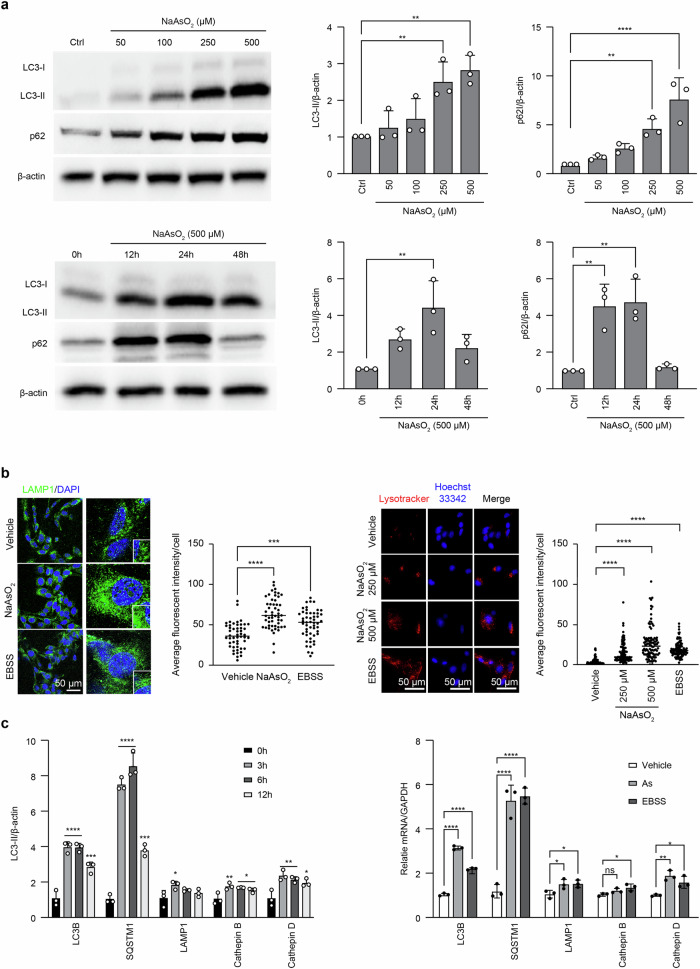
The reduced degradation capacity of the autophagy‒lysosome pathway affects the induction of premature senescence in HEI-OC1 cells subjected to a short exposure to NaAsO_2_. **a** The expression of LC3 and p62 at the protein level after a short exposure to NaAsO_2_ in HEI-OC1 cells (Right panel: dose-dependent change after exposure to 50, 100, 250, or 500 μM for 24 h; left panel: time-dependent changes after exposure to 500 μM for 12 h, 24 h, or 48 h) were measured by western blotting. Western blot analysis was performed as described in the “Materials and methods”. β-Actin was used as a loading control. The means ± S.D.s (fold changes compared to the control group) of three or more independent experiments are presented. **p < 0.01, Tukey’s test. Full-length blots are presented in Supplementary information Fig. S1C (left and right panels). **b** The fluorescence of LAMP1 (left panel) at 6 h after a short NaAsO_2_ exposure (250 μM for 1 h) and Lysotracker®Red DND-99 (right panel) at 6 h after a short NaAsO_2_ exposure (250 μM and 500 μM for 1 h). DAPI for LAMP1 and Hoechst 33342 for Lysotracker®Red DND-9 were used for nuclear staining. EBSS was used as a positive control. The intensity of LAMP1 and LysoTracker® Red DND-99 was also measured with ImageJ (NIH). All values are the means ± S.D.s from three or more independent experiments. ***p < 0.001, ****p < 0.0001, Tukey’s test. **c** Time course of the expression of autophagy-related genes (LC3B and p62) and lysosomal biogenesis-related genes (LAMP1, Cathepsin B, and Cathepsin D) in cells subjected to a short exposure to NaAsO_2_, measured by RT‒qPCR as described in the “Materials and methods”. GAPDH was used as a loading control. All values are the means ± S.D.s from three or more independent experiments. *p < 0.05, **p < 0.01, ***p < 0.001, ****p < 0.0001, ns: not significant, Tukey’s test.

Based on our previous report [19], we considered that oxidative stress-induced autophagy serves as a protective mechanism against premature senescence in auditory cells; we investigated the induction of LC3, which is a marker of autophagy, and p62, which turns over through autophagic degradation, and measured autophagic flux, an index of the autophagy degradation ability, in HEI-OC1 cells subjected to a short exposure to NaAsO_2_ to confirm whether autophagy is activated as a part of the DDR and regulates NaAsO_2_-induced cellular senescence in auditory cells. The expression of LC3-II and p62 was increased in a dose-dependent manner (Fig. 3a left panel) (Fig. S1C) and peaked at 24 h (Fig. 3a right panel) (Fig. S1C). As shown in Figs. S1D and S3A), the expression of LC3-II and p62 in bafilomycin A1 (BafA1)-treated cells was increased more at 24 h after a short exposure to NaAsO_2_ than after nonexposed cells. This result indicates that autophagic flux was promoted at 24 h after a short NaAsO_2_ exposure, which means that autophagic degradation activity occurs at this point in auditory cells.

Next, we considered the expression of lysosome-associated membrane protein 1 (LAMP1) and Lysotracker®Red DND-99, which are used as indicators of lysosomal abundance, via immunostaining in cells subjected to a short exposure to NaAsO_2_. LAMP1 is expressed on the surface of different organelles, including lysosomes, autolysosomes, endosomes, multivesicular bodies and multilamellar bodies (MVBs), in the cytoplasm [46, 47]. The fluorescence intensity of LAMP1 (Fig. 3b, left panel) was obviously elevated at 24 h after a short NaAsO_2_ exposure. It was higher than that of the EBSS-starved control. This result suggested that there might be LAMP1 positive-degradative and non-degradative organelles at this point in cells subjected to a short exposure to NaAsO_2_, which means there is an abundance of lysosomes.

According to previous reports, LysoTracker selectively accumulates in acidic organelles, including acidic vesicles, late endosomes, and lysosomes [48, 49]. Lysotracker®Red DND-99 (Fig. 3b, right panel) staining was also significantly increased 24 h after a short NaAsO_2_ exposure. This immunoexpression level was higher than that of EBSS, which was used as a positive control. This result indicated that lysosomal pH maintenance was compromised by a short NaAsO_2_ exposure. Namely, impaired organelles due to damaged lysosomes might accumulate in the cytoplasm of cells exhibiting auditory senescence at this point, with the lysosomal pH being maintained in an acidic environment.

Next, we investigated the time course of the TFEB-mediated gene expression of autophagy-related genes (LC3B and p62) and lysosome-related genes, including lysosome-associated membrane protein 1 (LAMP1) and representative proteases in lysosomes (Cathepsin B and Cathepsin D), in cells subjected to a short exposure to NaAsO_2_ by performing RT‒qPCR to evaluate the transcriptional function of TFEB and confirm the time-dependent changes in the autophagy-lysosomal pathway after a short exposure to NaAsO_2_.

As shown in Fig. 3c (left panel), the expression of autophagy-related genes was increased with a peak of 6 h, and that of lysosome-related genes was increased with a peak of 3 h. The expression level at 6 h was almost the same as that under EBSS starvation (Fig. 3c, right panel). Namely, the autophagy‒lysosome pathway (ALP) was activated at the early stage to protect auditory cells from NaAsO_2_-induced premature senescence, but it was only temporary, and the degradation capacity decreased soon after. These results indicate that the NaAsO_2_-induced impairment of autophagic degradation in senescent auditory cells is closely related to the reduced degradation capacity of the autophagy‒lysosome pathway (APL).

Next, we investigated the effect of TFEB on lysosomal function during premature senescence in auditory cells after a short exposure to NaAsO_2_. Lysosomal impairment induced by treatment with BafA1, which inhibits vacuolar ATPase (v-ATPase) on the lysosomal membrane, promotes alkalinization of the lysosomal lumen, and chloroquine (CQ), which elevates the lysosomal pH and inhibits lysosomal degradation, significantly increased the ratio of SA-βgal-positive cells among auditory cells after a short exposure to NaAsO_2_ by blocking the fusion of autophagosomes with lysosomes and impairing autophagic degradation through the autophagy‒lysosome pathway (APL) (Fig. S3B) [50, 51]. These results indicate that TFEB-associated lysosomal dysfunction directly affects the induction of premature senescence by blocking the fusion of autophagosomes with lysosomes in NaAsO_2_-exposed auditory cells.

We investigated the impact of TFEB on autophagy in auditory cells with premature senescence after a short exposure to NaAsO_2_. The mTOR inhibitor rapamycin obviously decreased the ratio of SA-βgal-positive cells among auditory cells after a short exposure to NaAsO_2_ by promoting autophagic degradation (Fig. S3C). This result means that the regulation of TFEB-mediated autophagy could directly affect premature senescence. Next, we examined ultrastructural premature senescence in NaAsO_2_-exposed auditory cells (Fig. S3D1–8). Importantly, ultrastructure analysis under TEM indicated that damaged mitochondria (Fig. S3D3–4) or dense organelles within immature autophagosomes, autolysosomes and multivesicular bodies (MVBs) or aggregates (Fig. S3D5–6) accumulated at 24 h in the cytoplasm of NaAsO_2_-exposed auditory cells [52]. Other autophagosomes eventually appeared to merge with lysosomes to become autolysosomes containing partially degraded material that appeared as electron-dense, unevenly distributed dense masses (Fig. S3D5–6). On the other hand, autophagosomes in EBSS-starved cells had a perfect shape with round, double-membraned structures (Fig. S3D7–8). This result means that the autophagy‒lysosome pathway (ALP) was defective at the ultrastructural level in NaAsO_2_-exposed auditory cells.

### TFEB nuclear translocation in auditory cells after exposure to NaAsO_2_ was only temporary

**Fig. 4:**
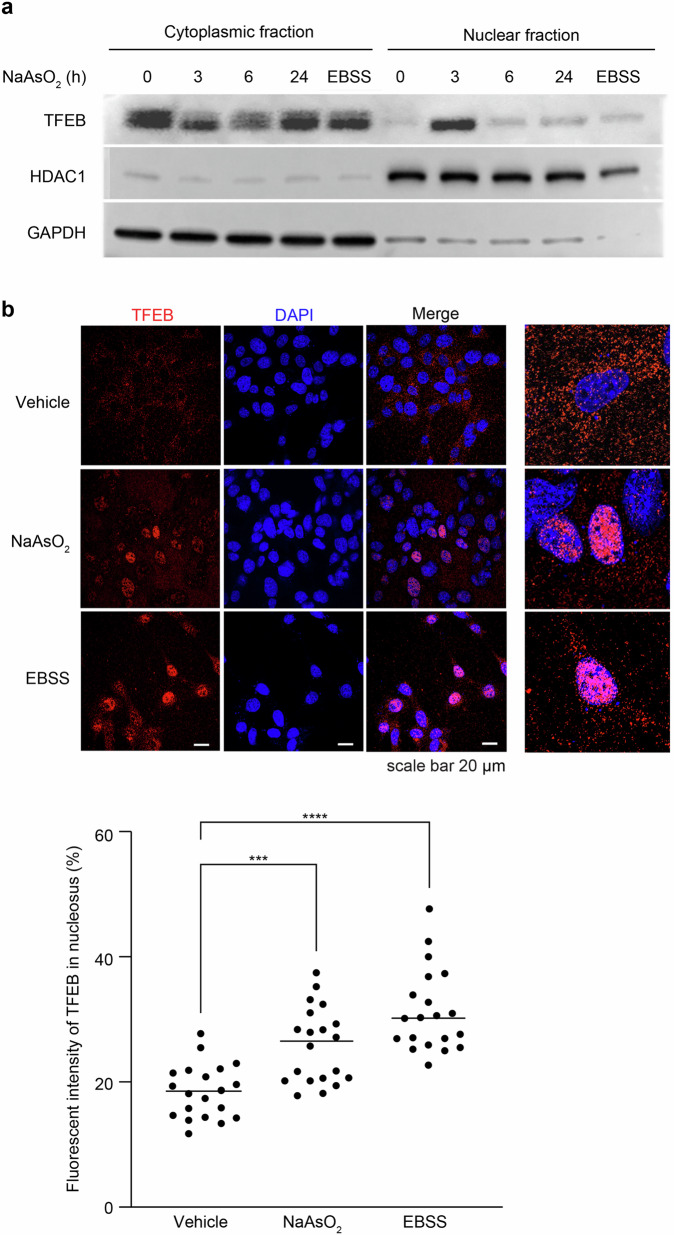
Short exposure to NaAsO_2_ temporarily activates TFEB, which translocates into the nucleus of auditory cells. **a** The temporal expression of TFEB in the nuclear and cytoplasmic fractions at 3 h after short exposure to NaAsO_2_ (500 μM for 1 h) in HEI-OC1 cells (right: nuclear fraction, left: cytoplasmic fraction) was measured by western blots. The translocation of TFEB at the same time (3 h) that it peaked in cells after NaAsO_2_ exposure was compared in a time-dependent manner with the translocation of TFEB in EBSS-starved cells. Western blot analysis was performed as described in the “Materials and methods”. GAPDH for the cytoplasm and HDAC1 for the nucleus were used as loading controls. All values are the means ± S.D.s from three or more independent experiments. *p < 0.001, **p < 0.0001. Full-length blots are presented in Supplementary information Fig. S1E. **b** The transportation of TFEB into nuclei from the cytoplasm at 3 h after a short NaAsO_2_ exposure (500 μM for 1 h) was detected by immunostaining. Nuclei were counterstained with DAPI. The peak expression of TFEB at 6 h in EBSS-starved cells was used as a positive control. Comparisons are made between vehicle, a short NaAsO_2_ exposure and EBSS treatment. The intensity of TFEB was measured by ImageJ (NIH). All values are the means ± S.D.s from three or more independent experiments. *p < 0.001, **p < 0.0001. Scale bars represent 20 μm in all images.

We focused on the function of transcription factor EB (TFEB), evaluating a TFEB nuclear export signal that activates autophagy and regulates lysosomal biogenesis in HEI-OC1 cells after a short exposure to NaAsO_2_ [28, 30, 53]. The expression of TFEB was increased at the peak of 3 h after a short exposure to NaAsO_2_ in the nuclear fraction but increased after 6 h in the cytoplasmic fraction (Fig. 4a and S1E, full-length blots), and the localization of TFEB in the nucleus was also significantly increased at 3 h (Fig. 4b). TFEB nuclear translocation was induced in a dose-dependent manner in cells subjected to a short exposure to NaAsO_2_ (Figs. S4 and S1F, full-length blots). These results indicate that endogenous TFEB in auditory cells is translocated into the nucleus from the cytoplasm and became temporarily activated until 3 h in a dose-dependent manner after a short exposure to NaAsO_2_ but subsequently declines.

### TFEB directly controls the induction of premature senescence by regulating the autophagy lysosomal pathway (ALP) and ROS derived from damaged mitochondria in HEI-OC1 cells subjected to a short exposure to NaAsO_2_

**Fig. 5:**
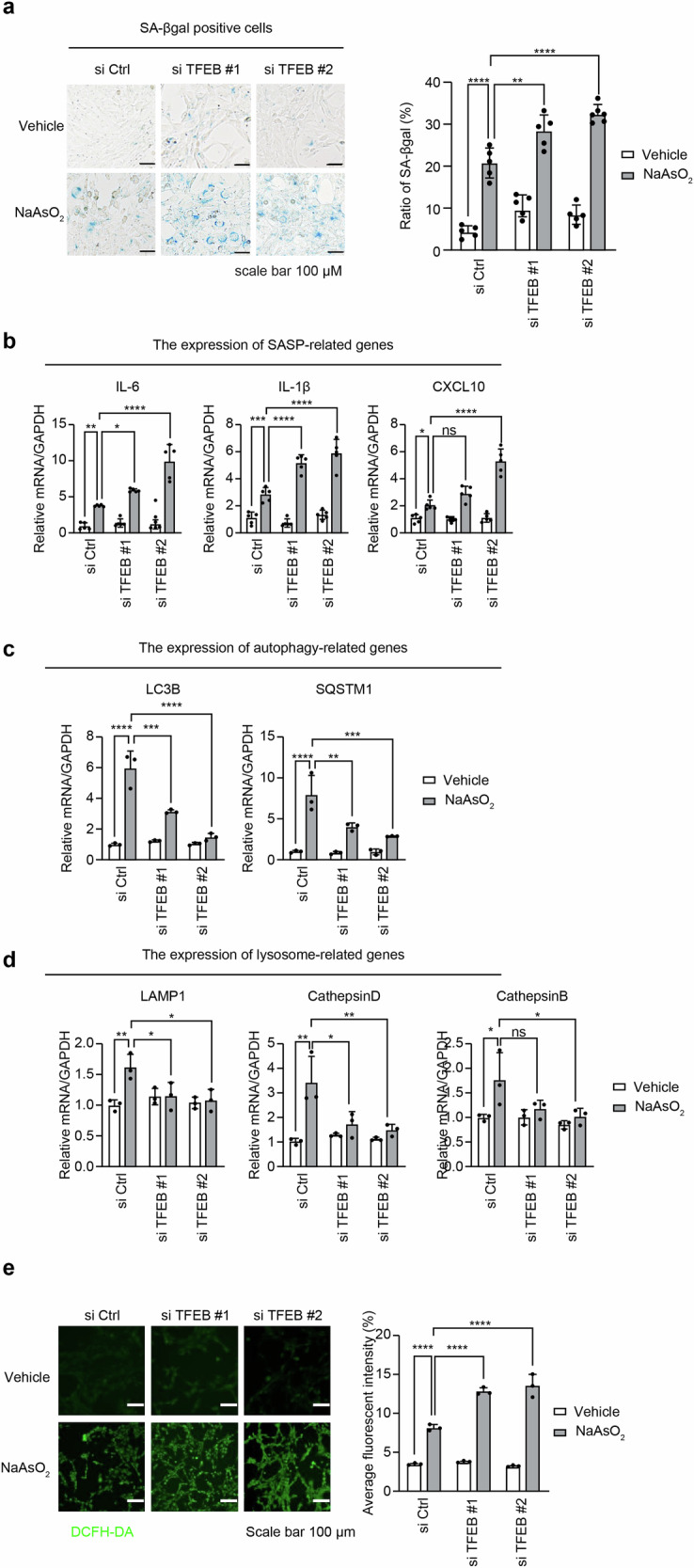
Crosstalk among TFEB, the autophagy lysosomal pathway (ALP) and ROS derived from damaged mitochondria in HEI-OC1 cells subjected to short exposure to NaAsO_2_ occurs. **a** The ratio of SA-βgal-positive cells in TFEB KD HEI-OC1 cells after short exposure to NaAsO_2_. After transfection with two TFEB (#1 and #2) and control (Ctrl) siRNAs for 48 h, the cells were exposed to NaAsO_2_ (500 mM for 1 h) as described in the “Materials and methods” and subjected to SA-βgal staining. SA-βgal-positive cells were quantified by counting more than 200 cells for each sample 3 days after this treatment, the positive rate of which was calculated from the ratio of positive cells to total cells. The control condition exhibited no detectable SA-β-gal staining. All values are the means ± S.D.s from three or more independent experiments. *p < 0.05, **p < 0.01, ***p < 0.001, ****p < 0.0001, ns: not significant, Tukey’s test. **b** The expression of SASP-related genes (IL-6, IL1, and CXCL10) in TFEB KD HEI-OC1 cells after short exposure to NaAsO_2_. The relative gene expression of IL-6, IL-1β, and CXCL10 was measured via RT‒qPCR 1 d after short-term NaAsO_2_ exposure. All values are the means ± S.D.s from three or more independent experiments. *p < 0.05, **p < 0.01, ***p < 0.001, ****p < 0.0001, ns: not significant, Tukey’s test. **c** The expression of TFEB target genes and autophagy-related genes (LC3B and p62) in TFRB KD HEI-OC1 cells. The relative mRNA expression of the LC3B and p62 genes was measured via RT‒qPCR 1 d after short NaAsO_2_ exposure. All values are the means ± S.D.s from three or more independent experiments. *p < 0.05, **p < 0.01, ***p < 0.001, ****p < 0.0001, ns: not significant, Tukey’s test. **d** The expression of TFEB target genes and lysosomal biogenesis-related genes (LAMP1, cathepsin B, and cathepsin D) in TFEB KD HEI-OC1 cells. The relative mRNA expression of genes (LAMP1, cathepsin B, and cathepsin D) was measured via RT‒qPCR 1 d after short NaAsO_2_ exposure. All values are the means ± S.D.s from three or more independent experiments. *p < 0.05, **p < 0.01, ***p < 0.001, ****p < 0.0001, ns: not significant, Tukey’s test. **e** The production of ROS in NaAsO_2_-shortly exposed TFEB (#1 and #2) KD cells. TFEB (#1 and #2) KD cells exposed to NaAsO_2_ (500 μM for 1 h) were incubated with DCFH-DA (10 μM for 1 h) and then visualized with fluorescence microscopy (KEYENCE) in EBSS, which was replaced after washing with PBS twice. *p < 0.05, **p < 0.01, ***p < 0.001, ****p < 0.0001, ns: not significant, Tukey’s test.

Finally, we knocked down TFEB expression using two different short interfering RNAs (siRNAs; #1: Santa Cruz Biotechnology, Inc., CA, USA and #2: Dharmacon Technologies, Lafayette, CO, USA) (Fig. S5) and evaluated the transcriptional function of TFEB during the induction of the premature senescence phenotype in HEI-OC1 cells after a short exposure to NaAsO_2_. Interestingly, the ratio of SA-βgal-positive cells (Fig. 5a) and the expression of SASP-related genes (IL-6, IL1, and CXCL10) (Fig. 5b) significantly increased in TFEB KD HEI-OC1 cells after a short exposure to NaAsO_2_. This result suggests that TFEB directly controls the induction of the premature senescence phenotype in cells subjected to a short exposure to NaAsO_2_.

Next, we investigated the transcriptional effects of TFEB-mediated targeting of autophagy-lysosomal pathway genes on premature senescence in auditory cells after a short exposure to NaAsO_2_. The expression of autophagy-related genes (LC3B and p62) (Fig. 5c) and lysosome-related genes (LAMP1, cathepsin B, and cathepsin D) (Fig. 5d) was significantly decreased in TFRB KD cells. The autophagy-related proteins (LC3-II and p62) and lysosomal protein (active cathepsin B) was also not induced in TFEB KD cells after a short exposure to NaAsO_2_ (Fig. S6 and S1G (full-length blots)). This result indicates that TFEB promotes autophagy and lysosomal biogenesis in auditory cells, regulating the transcription of autophagy-related genes (LC3B and p62) and lysosome-related genes (LAMP1, cathepsin B and cathepsin D) at the transcriptional level.

As shown in Fig. 5a–d, the induction of a premature senescence phenotype and the expression of TFEB target genes were negatively correlated in auditory cells after a short exposure to NaAsO_2_. These results suggested that TFEB targeting ALP dysfunction leads to premature senescence in auditory cells subjected to a short exposure to NaAsO_2_.

As shown in Figs. S7 and S1Ｆ (full-length blots), NAC substantially decreased the expression of TFEB in the nuclear fraction but increased TFEB expression in the cytoplasmic fraction. Interestingly, the knockdown of TFEB via siRNA significantly increased the production of ROS from damaged mitochondria in NaAsO_2_-exposed cells (Fig. 5e). These two results mean that the TFEB export signal is dependent on ROS production derived from damaged mitochondria, controlling mitochondrial quality.

## Discussion

In this study, we confirmed that TFEB might function as a master regulator of the induction of oxidative stress-induced premature senescence and the senescence-associated secretory phenotype (SASP) in auditory cells, regulating the autophagy-lysosomal pathway (ALP) and controlling ROS production derived from damaged mitochondria. Our results suggested that a short NaAsO_2_ exposure leads to DNA damage, lysosomal damage and mitochondrial damage in auditory cells, triggering temporary TFEB transport into the nucleus and as a result, causing insufficient autophagic flux (autophagic degradation) and declines in lysosomal function and biogenesis and mitochondrial quality. Then, the intracellular ROS derived from damaged mitochondria play a role as a second messenger in inducing premature senescence in auditory cells. These findings suggest that TFEB activation via transport into the nucleus might contribute to the anti-senescence activity of auditory cells as a new therapeutic target for ARHL.

First, we established a NaAsO_2_-induced premature senescence model of ARHL to clarify the role of the autophagy degradation system in senescent auditory cells. An important aspect of our model is that extracellular NaAsO_2_ exposure leads to simultaneous DNA damage and lysosomal and mitochondrial damage in cells, inducing premature senescence and autophagy. As reported in a previous study [54, 55], DNA damage induces the DDR and autophagy, which are two important processes for maintaining cellular homeostasis. Importantly, autophagy is thought to be required for the functional outcomes of DDR signaling, including premature senescence and the SASP, to maintain genomic stability [56]. Recent studies have demonstrated that DNA damage induces autophagy by activating autophagy-related genes via checkpoint kinase 2 (CHK2)–FOXK (FOXK1 and FOXK2) axis-mediated transcriptional control [57] and that the DDR induces senescence and SASP by inhibiting autophagy of the transcription factor GATA4 [16]. However, it remains unclear how premature senescence and SASP are ultimately induced in auditory cells by bypassing the intracellular clearance system of autophagy. In this study, we confirmed induction of the DDR on the basis of the phosphorylation of H2AX and DNA damage-induced senescence on the basis of the increased SA-βgal-positive cell numbers and expression of the SASP. Our data demonstrated that NaAsO_2_-exposed cells have the ability to promote the autophagic degradation pathway (autophagic flux); however, the inducible expression of LC3-II and p62 was only temporary, and dense organelles within the incomplete autolysosome or autolysosome were confirmed in NaAsO_2_-exposed auditory cells. These results indicate that the status of autophagic flux is incomplete or immature in auditory cells with NaAsO_2_-induced senescence. Accumulating evidence shows that the restoration of autophagic flux could be a potential target for antiaging therapeutics [27, 58]. Based on these previous studies, we focused on the function of the autophagy-lysosomal pathway (ALP) in our senescent auditory cell model. Then, we found that two lysosomal function-dependent autophagy inhibitors, BafA1 and CQ, promoted premature senescence, while the mTOR inhibitor rapamycin suppressed premature senescence. CQ inhibits the fusion of autophagosomes and lysosomes progressively without substantially changing lysosomal acidity [51]. BafA1 prevents functional autophagic flux and cargo degradation by inhibiting lysosomal proton pump V-ATPase-dependent acidification and blocking Ca-P60A/SERCA-dependent autophagosome-lysosome fusion [59]; this decreases lysosomal degradation capacity and disturbs mTOR signaling in this organelle [60, 61]. On the other hand, CQ blocks autophagosome-lysosome fusion without affecting the degradation capacity of lysosomes, leading to an accumulation of autophagosomes rather than nonfunctional autolysosomes [62]. Importantly, these results suggested that premature senescence in auditory cells with NaAsO_2_-induced senescence might be caused by blocking autophagosome-lysosome fusion. Previous reports also indicated that the activation of lysosomal function in the autophagic pathway depends on a dual mechanism involving mTORC1 inhibition and autophagosome-lysosome fusion [63]. On the other hand, the inhibition of mTORC1 by rapamycin promoted the activation of lysosome function and autophagosome-lysosome fusion in auditory cells with NaAsO_2_-induced senescence, reducing premature auditory senescence. Specifically, we showed that lysosomal activation and the promotion of autophagosome-lysosome fusion play keys roles in the regulation of NaAsO_2_-induced auditory premature senescence.

As reported in a previous study, the source of cellular ROS is mainly mitochondria, and a low mitochondrial membrane potential (MMP) increases the production of reactive oxygen species (ROS) [64]. A recent report suggested that the dysregulation of a selective type of macroautophagy called mitophagy leads to the excessive production of ROS, reducing the ability to remove damaged mitochondria; the resulting imbalance among mitophagy, ROS production, and mitochondrial damage can initiate and accelerate the aging process [65]. Mitochondrial dysfunction-associated ROS are produced during stress-induced senescence [66]. Based on these previous reports, we focused on the function of ROS as potential secondary messengers that could be used to specifically modulate distinct cellular pathways [67]. In fact, we obtained very interesting results that NAC treatment reduced the percentage of SA-βgal-positive cells and the expression of SASP-related genes, suppressing the expression of p21 and decreasing the production of intracellular ROS, although the number of γH2AX foci/cell was not affected. This result means that there is a different pathway from DDR signaling and that intracellular ROS from damaged mitochondria serve as a second messenger for triggering the premature senescence phenotype in auditory cells. Previous studies have also noted that ROS can modify putative target protein activity or conformation via the reversible oxidation of specific cysteine or methionine residues within redox-sensitive proteins, altering signal transduction and contributing to aging-related mechanisms, inflammation, and autophagy. From these reports, we consider that there should be a feedback loop between p21 and ROS production for inducing premature senescence in auditory cells with NaAsO_2_-induced senescence [68].

Importantly, we confirmed that LC3B and p62 among autophagy-related genes and LAMP1, Cathepsin B and D among lysosome-associated genes are included among TFEB target genes in auditory cells, which means that TFEB inactivation leads to a decrease in autophagic flux and lysosomal dysfunction via the transcriptional response of lysosomal and autophagic genes. As a consequence, an insufficient ALP promotes premature auditory senescence. In addition, staining of Lamp1 and LysoTracker was abundant under a light microscope in the cytoplasm of NaAsO_2_-exposed cells. This result suggested that dysfunctional lysosomes or endosomes might increase due to impaired lysosomal biogenesis, leading to an incomplete ALP and auditory senescent cells with a lowered pH. Our study was the first report to indicate the critical role of TFEB downregulation in ALP insufficiency in NaAsO_2_-induced premature auditory senescence. Surprisingly, the number of SA-βgal-positive cells and the expression of SASP-related genes showed a clear inverse correlation with the decreased expression of autophagy- and lysosome-related genes but were correlated with increased ROS production in NaAsO_2_-exposed TFEB KD auditory cells. These results suggested that TFEB might regulate autophagic flux at the transcriptional level by promoting the fusion between autophagosomes and lysosomes in the ALP and controlling mitochondrial quality in auditory cells with NaAsO_2_-induced senescence. A previous study showed that TFEB activation contributes to the clearance of ROS and resistance to oxidative stress [69], promotes lysosomal biogenesis and function, and controls autophagic proteins, eliminating ROS [70]. Some reports have also indicated that TFEB protects cells from oxidative stress via autophagy either in vivo or in vivo [71, 72]. Interestingly, our data demonstrated that NAC treatment completely suppressed the expression of TFEB in NaAsO_2_-exposed auditory cells. This result suggested that the ROS-dependent TFEB activation pathway exists in NaAsO_2_-exposed auditory cells. According to a recent study, there is accumulating evidence that cellular ROS can induce rapid nuclear translocation of TFEB, but neurodegenerative disorders are characterized by excess ROS [73]. Overall, ROS from damaged mitochondria might function as a second messenger for the first nucleocytoplasmic shuttling of TFEB in auditory cells, but accumulating ROS cause partial impairment of the autophagy lysosomal pathway, leading to premature auditory senescence.

Consistent with our results, it has been reported previously that the impairment of TFEB promotes mitochondrial ROS production, while the activation of TFEB with gemfibrozil, an activator of peroxisome proliferator-activated receptor-alpha (PPARα), which is usually used for the treatment of hypercholesterolemia, leads to the induction of autophagy and reductions of oxidative stress [74]. Furthermore, TFEB could regulate mitophagy by interacting with PINK1/Parkin, reducing ROS [75]. Specifically, TFEB may control mitochondrial quality through selective autophagy and mitophagy, in auditory cells, regulating ROS production. We conclude that there is crosstalk among TFEB, the autophagy lysosomal pathway (ALP) and ROS derived from damaged mitochondria in auditory cells under oxidative stress (NaAsO_2_).

**Fig. 6:**
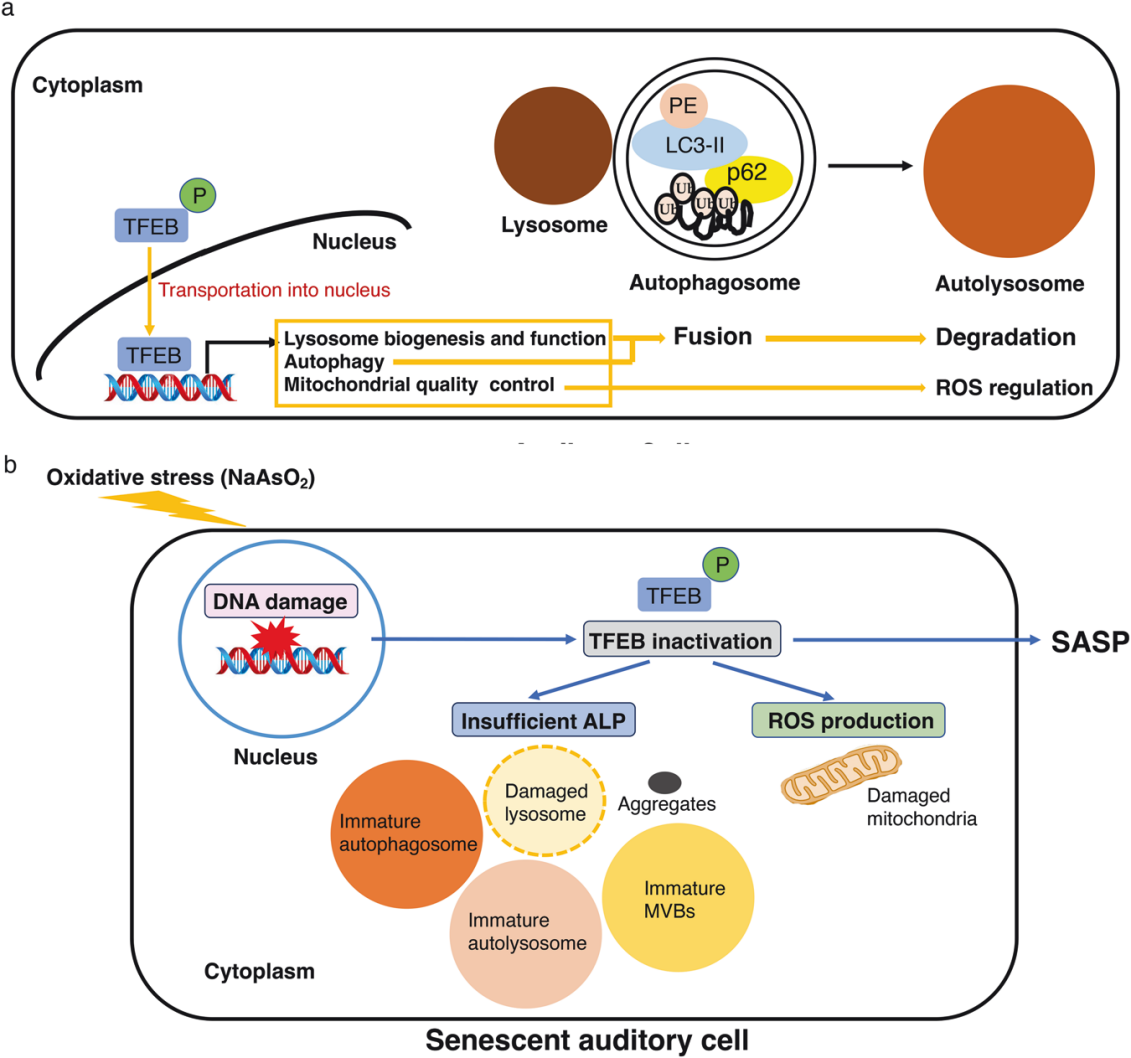
The mechanism of TFEB-mediated regulation of oxidative stress-induced premature senescence in auditory cells. **a** TFEB may regulate autophagic flux by promoting the fusion of autophagosomes and lysosomes in the ALP and controlling mitochondrial quality in auditory cells. **b** The inactivation of TFEB by oxidative stress (NaAsO_2_) directly causes immature autophagosomes, autolysosomes and multivesicular bodies (MVBs) or aggregates via decreases in autophagic flux and lysosomal dysfunction, with decreased pH levels and ROS production due to decreased mitochondrial quality in auditory cells, inducing premature auditory senescence and SASP induction.

In conclusion, this is the first report to indicate that the inactivation of TFEB directly causes oxidative stress (NaAsO_2_)-induced premature auditory senescence and SASP induction via decreases in autophagic flux and lysosomal dysfunction, with a lowered pH at the transcriptional level and, as a consequence, ROS production with decreasing mitochondrial quality in auditory cells, as shown in Fig. 6. Our data suggested that TFEB could be a therapeutic target for preventing ARHL and that the activator of TFEB might have a pivotal antiaging effect in the inner ear.

## Materials and methods

### Cell line and culture conditions

The HEI-OC1 cell line was kindly provided by F. Kalinec (University of California, Los Angeles, Los Angeles, CA, USA). The establishment and characterization of this conditionally immortalized mouse auditory cell line have been described previously [76, 77]. Cells were maintained in high-glucose Dulbecco’s modified Eagle’s medium (DMEM; Gibco BRL, Grand Island, NY, USA) containing 10% fetal bovine serum (FBS; HyClone, UT, USA), 1% penicillin-streptomycin, and 1% kanamycin (MP Biomedicals, Ohio, USA). HEI-OC1 cells were cultured under the following permissive conditions: 33 °C and 5% CO2 in DMEM supplemented with 10% FBS. Cells were grown to 80% confluency, and regular medium was exchanged for medium with or without drugs.

### Cell viability assay

HEI-OC1 auditory cells (2 × 10^4^ cells/ml/well in 12-well plates) were incubated with culture medium supplemented with 50 mM NaAsO_2_ solution so that the final concentrations were 125 μM, 250 μM and 500 μM for 1 h and then cultured under permissive conditions for 3, 6, 12, 24 and 48 h with complete culture medium lacking NaAsO_2_, which was replaced after washing with PBS two times at 33 °C. Control cells were also prepared similarly without NaAsO_2_. To determine cell viability, the cells were washed with phosphate-buffered saline (PBS), harvested from the dish via trypsinization (0.05% trypsin, 0.53 mM EDTA for 2 min), resuspended in PBS, and diluted 1:1 in 0.4% trypan blue solution. Cell viability was defined as the percentage of live cells among total (dead plus live) cells, and the number of live cells was calculated with a TC10 Automated Cell Counter (Bio-Rad, USA). All experiments were performed in triplicate.

### Reagents and antibodies

Sodium arsenite (NaAsO_2_), chloroquine diphosphate salt (CQ), and N-acetyl-L-cysteine (NAC) were purchased from Sigma-Aldrich (St. Louis, MO, USA). Bafilomycin A1 (BafA1) and rapamycin (Rap) were purchased from Selleck (Houston, USA). The following primary antibodies for western blot analysis and immunostaining were purchased from Cell Signaling Technology (Danvers, MA, USA): anti-TFEB, anti-p62/SQSTEM1, anti-gH2AX, anti-cathepsin B, anti-HDAC1 and anti-GAPDH antibodies. The anti-p16, anti-p21, and Alexa488-conjugated anti-LAMP1 antibodies were obtained from Santa Cruz Biotechnology, Inc. (Dallas, TX, USA). The anti-LC3 antibody was purchased from MBL. Anti-β-actin was purchased from Dako (Glostrup, Denmark). The following secondary antibodies were from Cell Signaling Technology (Danvers, MA, USA): HRP-conjugated anti-mouse and anti-rabbit antibodies and anti-rabbit IgG antibody (Alexa Fluor^®^ 555 Conjugate). Lysotracker® Red DND-99 was purchased from Thermo Fisher Scientific (Waltham, MA, USA). Small interfering RNA (siRNA) for TFEB #1 and control siRNA were from Santa Cruz Biotechnology, Inc., and TFEB #2-siRNA was from Dharmacon Technologies (Lafayette, CO, USA). The E-PER Nuclear and Cytoplasmic Extraction Reagents for investigating the nuclear fraction and cytoplasmic fraction were from Thermo Scientific (Waltham, MA, USA).

### Protein extraction and western blot analysis

The cells were washed three times with ice-cold PBS and then lysed in RIPA lysis buffer containing protease inhibitor cocktails (final concentrations: 10 mM NaF, 1 mM sodium orthovanadate, 1 mM PMSF, 1× Complete Mini (Roche, Mannheim, Germany), 0.3 μg/mL Trichostatin A, 10 mM nicotinamide, 5 mM beta-glycerophosphoric acid, 1 mM dithiothreitol (DTT)) for 30 min on ice. Insoluble fractions of cell lysates (2–3 mg) were removed by centrifugation at 13,000 × *g* at 4 °C for 5 min and denatured by boiling in EzApply (AE-1430; ATTO Co, Japan). The protein concentrations were determined using a Pierce BCA Protein Assay Kit (Thermo Fisher Scientific, Inc., Waltham, MA, USA).

The samples (15-30 μg) were subjected to electrophoresis on sodium dodecyl sulfate-polyacrylamide gels (SDS‒PAGE; SDG-575, BIO CRAFT) (5–20%) for 90–120 min at 20 mA and then transferred onto PVDF membranes using iBlot (Life Technologies, Carlsbad, CA, USA). Following blocking with 5% nonfat milk (Amersham ECL blocking agent, GE Healthcare) at room temperature for 1 h, the membrane was incubated overnight at 4 °C in the presence of primary antibodies at dilutions of 1:1000–1:3000 in TBS-T. After three washes with TBS-T, the membrane was incubated with the corresponding species-appropriate secondary antibodies at a dilution of 1:2000–1:3000 in TBS-T for 1 h. Then, the immunoreactive bands on the membrane were visualized using a LAS-4000 mini (Fujifilm, Tokyo, Japan). Detection was carried out using a chemiluminescent imaging system (ImageQuant LAS 4010, UK) after incubation with SuperSignal^TM^ West Femto Maximum Sensitivity Substrate (Thermo, USA) according to the manufacturer’s instructions. All blots were derived from the same experiment and processed in parallel.

### Total RNA isolation and quantitative RT‒PCR analysis

Total RNA was isolated with an RNeasy Mini Kit (QIAGEN, Hilden, Germany) according to the manufacturer’s instructions. HEI-OC1 cells were cultured on 12-well plates for 24 h, and 600 μl of cell lysis buffer was added after washing with PBS one time. The cells were retrieved in 1.5 ml tubes after vortexing for 1 min, stirred by pipetting after adding 600 μl of 70% ethanol and centrifuged into RNeasy spin columns at 13,500 rpm for 15 s. Then, total RNA was eluted from the treated cells into a new RNeasy spin column in which 30 μl of RNase-free water was applied by centrifugation at 13,500 rpm for 1 min as described previously. The concentration and degree of purity of the extracted tRNA was measured on the basis of the OD_260_ and OD_280_ values using a NanoDrop Lite (Thermo Fisher Scientific, USA).

Complementary DNA (cDNA) was generated using the ImProm-II Reverse Transcription system (Promega Corporation, Madison, WI) according to the manufacturer’s instructions. Total RNA (250-500 μl) was mixed with oligo(dT) primer and RNase-free water. This reaction mixture (5 μl) was incubated at 70 °C for 5 min in a thermal cycler and then cooled for 5 min. ImProm-II 5 x Reaction buffer (2 μl), 25 mM MgCl_2_ (1.2 μl), 10 mM deoxynucleotide triphosphate mixture (0.5 μl) and ImProm-II Transcriptase (0.5 μl) were mixed with RNase-free water to a final volume of 10 μl. The cDNA elongation reaction was performed by incubation at 42 °C for 60 min in a thermal cycler after annealing the oligo dT primer and mRNA by incubating the reaction liquid 10 μl at 25 °C for 5 min in a thermal cycler. For cDNA synthesis, the isolated cDNA, which was subjected to enzyme via incubation at 70 °C for 15 min, was held on ice and then diluted three times with pure water. The real-time (RT)-PCR data collection was performed with an ABI PRISM 7000 Sequence Detection System (Applied Biosystems, Foster City, CA). Then, 10 μM forward primer (0.3 μl) and 10 μM reverse primer (0.3 μl) were mixed with cDNA (10 μl), PowerUp SYBR Green Master Mix (Applied Biosystems, Darmstadt, Germany) (7 μl) and ultrapure water (4.9 μl) to a total volume of 15 μl. The sequencing primers are listed in Table S1. The following experimental protocol was used: heating (95 °C for 15 s), denaturation program (95 °C for 15 s), and annealing and elongation (60 °C for 60 s) repeated 40 times for amplification. Data collection was performed using a QuantStudio 7 Flex Detection System (Thermo Fisher Scientific, USA). The mRNA expression level of each gene was normalized using glyceraldehyde 3-phosphate dehydrogenase (GAPDH) as an internal control.

### Senescence-associated β-galactosidase (SA-βgal) staining

SA-βgal staining in HEI-OC1 cells was performed using a Senescence β-Galactosidase Staining Kit (#9860, Cell Signaling Technology). As described above, HEI-OC1 cells were incubated with NaAsO_2_ at a final concentration of 250 μM or 500 μM for 1 h and then cultured with complete culture medium lacking NaAsO_2_, which was replaced at 33 °C for 72 h after washing with PBS once. The cells were cultured on 6-well plates and then fixed with Fixative Buffer for 10 min after washing with PBS one time. The cells were then incubated with 1 ml of staining buffer containing X-Gal substrate adjusted to pH 7 to 8 after washing with PBS overnight at 37 °C in a humidified incubator without the added 10% CO_2_ mixture (i.e., 100% ambient room air). Cells were observed under an inverted fluorescence phase-contrast microscope (BioZero BZ-8100 All-In-One Fluorescence Microscope, Keyence, Osaka, Japan). Cells with blue staining in the cytoplasm were scored as positive, and more than 200 cells were counted for each sample in each experiment to quantify positive staining.

### Immunofluorescence and quantification

Immunostaining for γH2AX, LAMP1, Lysotracker®Red DND-99 and TFEB was performed in the cytoplasm of HEI-OC1 cells. Cells grown on 35 mm glass-bottom dishes (Greiner, Germany) or coverslips were fixed with 4% PFA in PBS at room temperature for 1 h, permeabilized with 0.1% Triton X-100 for 5 min and then incubated with 0.1% (v/v) Tween 20 (PBST) blocking buffer at room temperature for 1 h. The cells were then incubated with primary antibodies in staining buffer (1% BSA and 0.05% saponin in PBS) at 4 °C overnight. The dilutions of individual antibodies are listed in Tables S2 and S3. Cells were washed three times in PBS and incubated with secondary antibodies for 1 h at room temperature in a dark room. The cells were washed with PBS three times, and then nuclei in the cells were stained with mounting medium containing 4’,6-diamidino-2-phenylindole (DAPI) (VECTSSHIELD Mounting Medium, Vector Laboratories). All samples were visualized under an inverted fluorescence phase-contrast microscope (BioZero BZ-8100 All-In-One Fluorescence Microscope, Keyence, Osaka, Japan) and counted under an inverted fluorescence phase-contrast microscope (BioZero BZ-8100 All-In-One Fluorescence Microscope, Keyence, Osaka, Japan) for γH2AX foci and Lysotracker®Red DND-99 or an inverted confocal microscope (A1R, Nikon, Tokyo, Japan) for LAMP1 and TFEB foci. The number of γH2AX foci per cell was quantified with ImageJ (National Institutes of Health (NIH); http://imagej.nih.gov/ij/). The intensity of LAMP1, TFEB and Lysotracker®Red DND-99 staining was also measured with ImageJ (NIH).

### Measurement of intracellular ROS generation with DCFH-DA

Intracellular ROS generation was measured with DCFH-DA (2’,7’-dichlorodihydrofluorescin diacetate, Sigma). HEI-OC1 cells, which were cultured in 12-well dishes and treated as shown here, were incubated with 10 μM DCFH-DA for 1 h and then visualized with fluorescence microscopy (KEYENCE); the cells were visualized in EBSS, which was replaced after washing with PBS twice.

### Evaluation of intracellular lysosome activity with LysoTracker® Red DND-99

Intracellular lysosome activity was evaluated with Lysotracker®Red DND-99 (Thermo Fisher Scientific). Cells were grown on 35 mm glass-bottom dishes (Greiner, Germany) for 24 h and incubated with medium (DMEM with 10% FBS) supplemented with LysoTracker® Red DND-99 (Thermo Fisher Scientific) at a final concentration of 50 nM at 37 °C for 1 h. The cells were washed with PBS three times, and then nuclei in cells were stained with PBS containing Hoechst 33342 (10 μg/ml). The cells were observed under an inverted fluorescence phase-contrast microscope (BioZero BZ-8100 All-In-One Fluorescence Microscope, Keyence, Osaka, Japan). Cells with red staining puncta in the cytoplasm that had an intensity higher than the threshold were scored as positive, and more than 100 cells were counted with ImageJ (http://imagej.nih.gov/ij/) for each sample in each experiment to quantify positive staining.

### Transmission electron microscopy

The cells were fixed with 2.5% glutaraldehyde in 0.1 M cacodylic buffer solution (pH 7.4) overnight, postfixed with 1% osmium tetroxide in 0.1 M cacodylic buffer solution (pH 7.4) for 2 h, dehydrated through graded ethanol, and embedded in Quetol-812. Ultrathin sections were cut with a diamond knife on an ultramicrotome (ULTRACUT UCT, Leica, Wien, Austria). The thin sections were stained with uranyl acetate and lead citrate and observed using a transmission electron microscope (JEM-1200EX, JEOL, Tokyo, Japan).

### Transient siRNA transfection

Two small interfering RNAs (siRNAs), *TFEB #1* (Santa Cruz Biotechnology, Inc.) and *TFEB #2* (Dharmacon Technologies, Lafayette, CO, USA), were used for knockdown of the *TFEB* gene, and control siRNA (Santa Cruz Biotechnology, Inc.) was used as a control. HEI-OC1 cells (2 × 10^4^ cells/ml/well in 6-well plates) were cultured overnight to achieve 90–95% confluency. Each siRNA (60 pmol) and the Lipofectamine® RNAiMAX Reagent (Thermo Fisher Scientific) (4.5 μl) were mixed with 120 μl of Opti-MEM/reduced serum medium (Thermo Fisher Scientific) for transfection. This mixture was incubated at room temperature for 15 min and then added to the medium, with which the cells were cultured for 48 h. Two days after transfection, the cells were either treated or remained untreated and then prepared for SA-βgal staining, measurement of intracellular ROS generation with DCFH-DA, western blotting or qPCR analysis, as described in the “Materials and methods”.

### Statistical analysis

Statistical analysis was carried out with Prism9 (GraphPad). All the data are expressed as the mean ± S.D. Student’s *t* test was applied for comparisons between two groups, and one-way variance (ANOVA) was used for comparisons among more than three groups. Tukey’s multiple comparison test was applied if there was a significant difference between them. A *p* value less than 0.01 (*) or 0.001 (**) indicated statistical significance.

### Supplementary information

Figure S1 full length blot

Figure S2

Figure S3

Figure S4

Figure S5

Figure S6

Figure S7

Supplementary Table S1-3

Supplementary figure legends

## Data Availability

All data and information related to this study will be made available from the corresponding authors upon request.
