# Supplementary figures and images for: Premature senescence is regulated by crosstalk among TFEB, the autophagy lysosomal pathway and ROS derived from damaged mitochondria in NaAsO2-exposed auditory cells
Source: Cell Death Discov. 2024 Aug 28;10:382. doi: 10.1038/s41420-024-02139-4 (PMC11350138; doi:10.1038/s41420-024-02139-4)

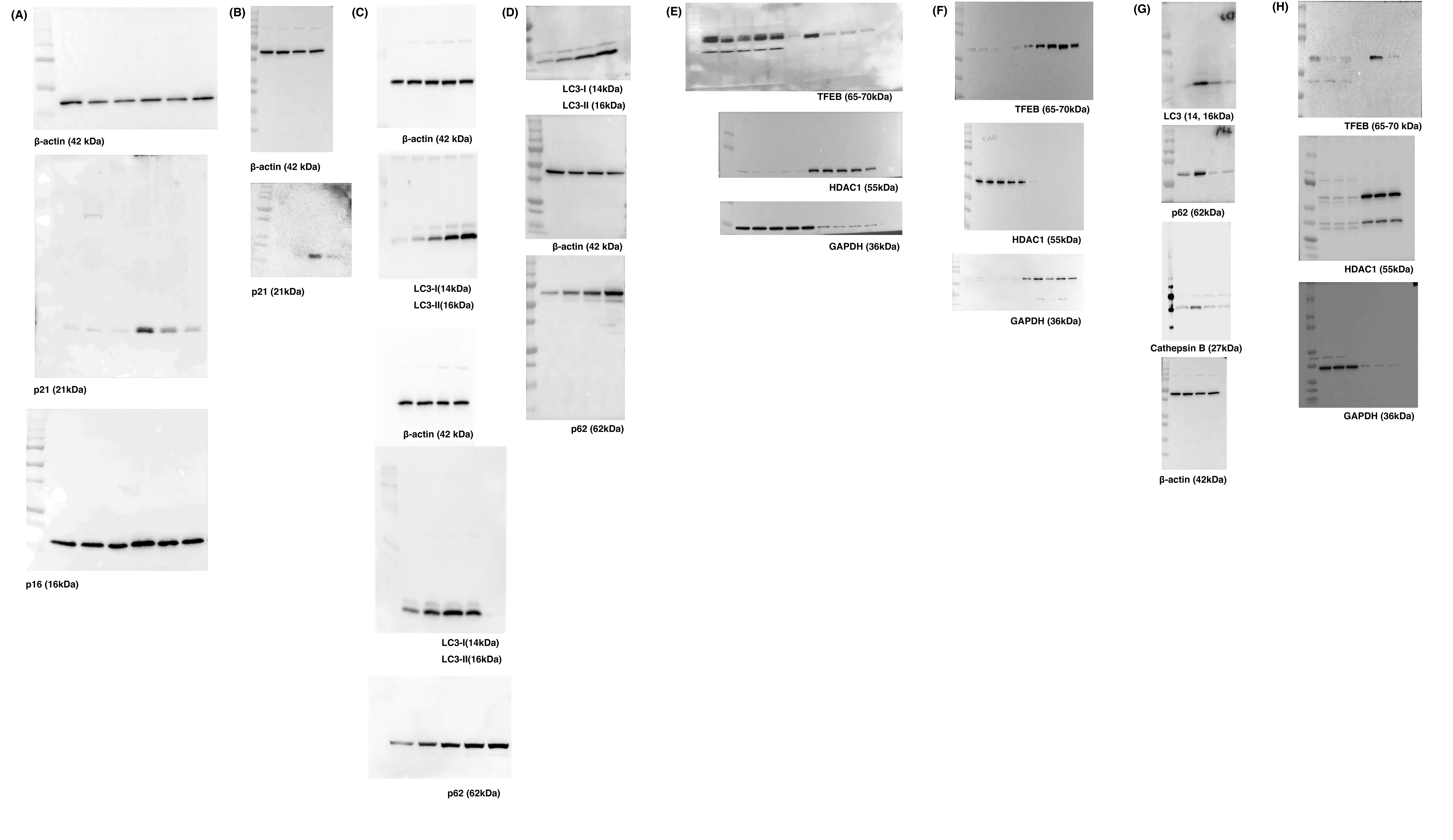

Supplement: Supplementary file 1 — Figure S1 full length blot [file 41420_2024_2139_MOESM1_ESM.tif]

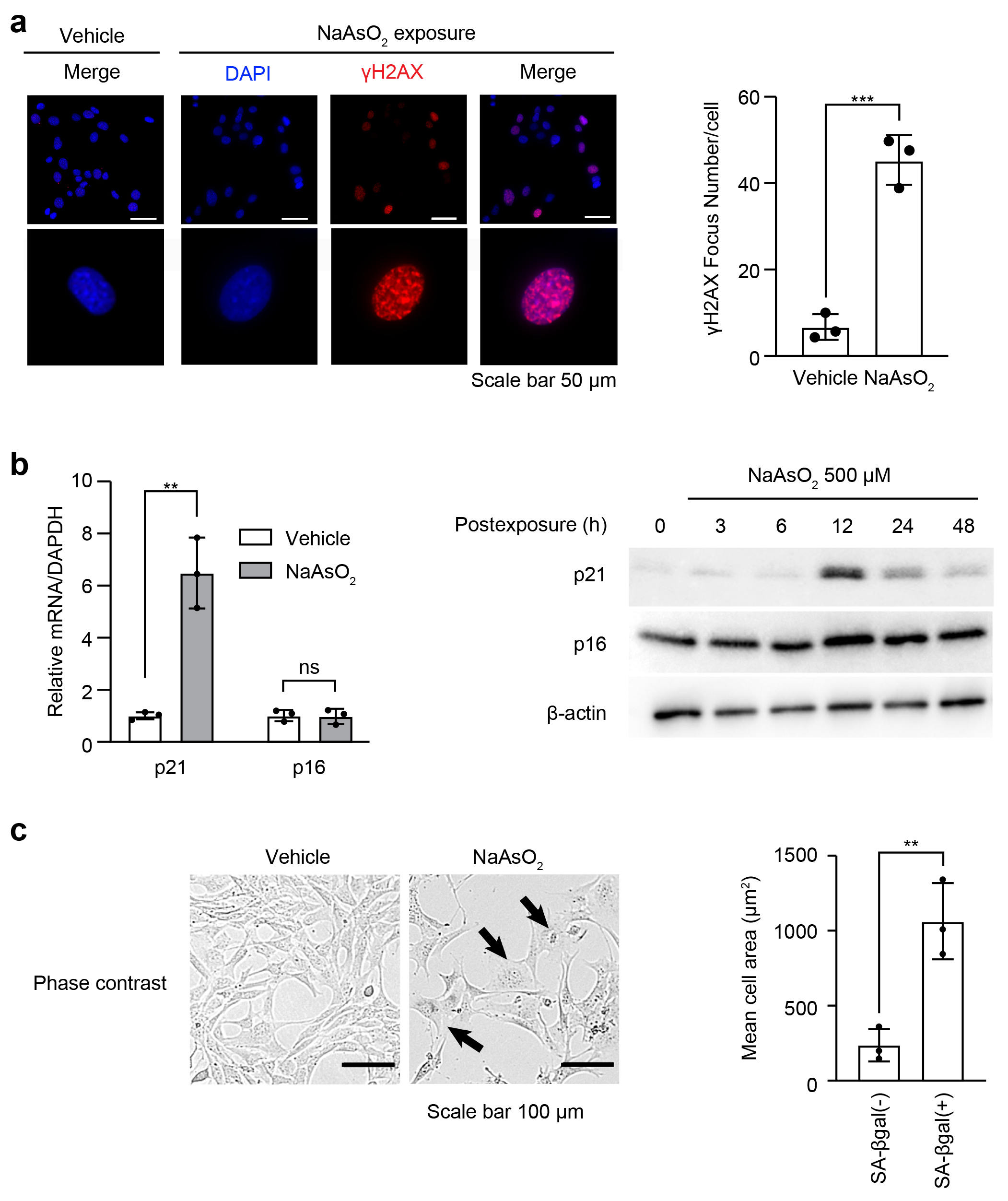

Supplement: Supplementary file 2 — Figure S2 [file 41420_2024_2139_MOESM2_ESM.tif]

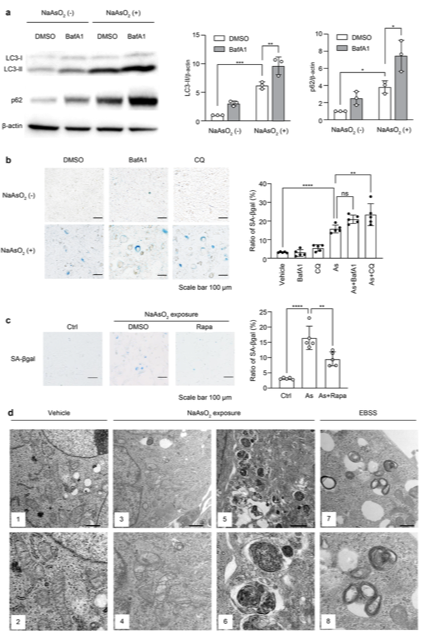

Supplement: Supplementary file 3 — Figure S3 [file 41420_2024_2139_MOESM3_ESM.tiff]

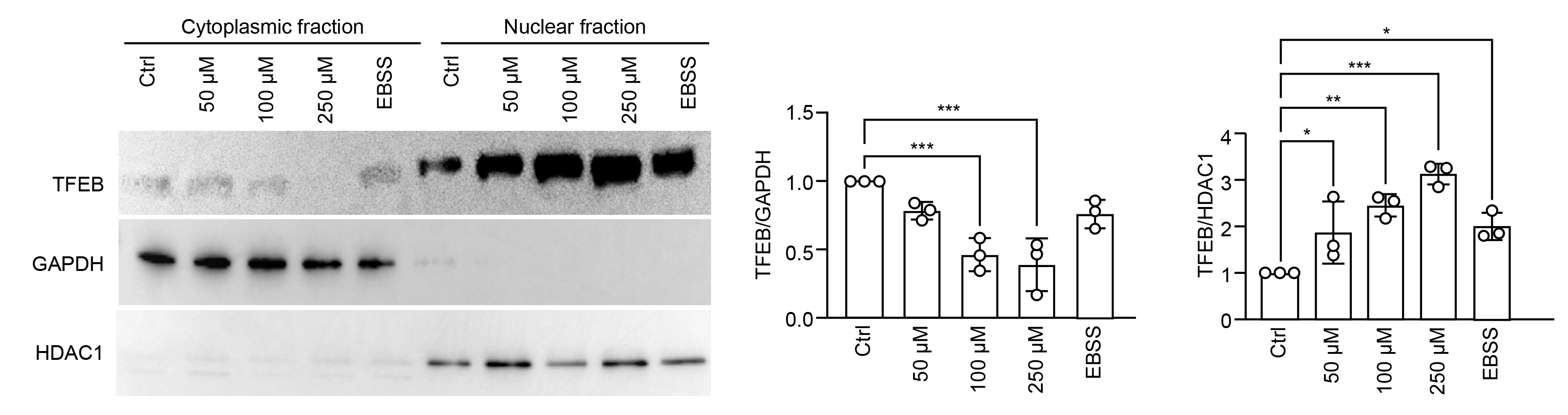

Supplement: Supplementary file 4 — Figure S4 [file 41420_2024_2139_MOESM4_ESM.tif]

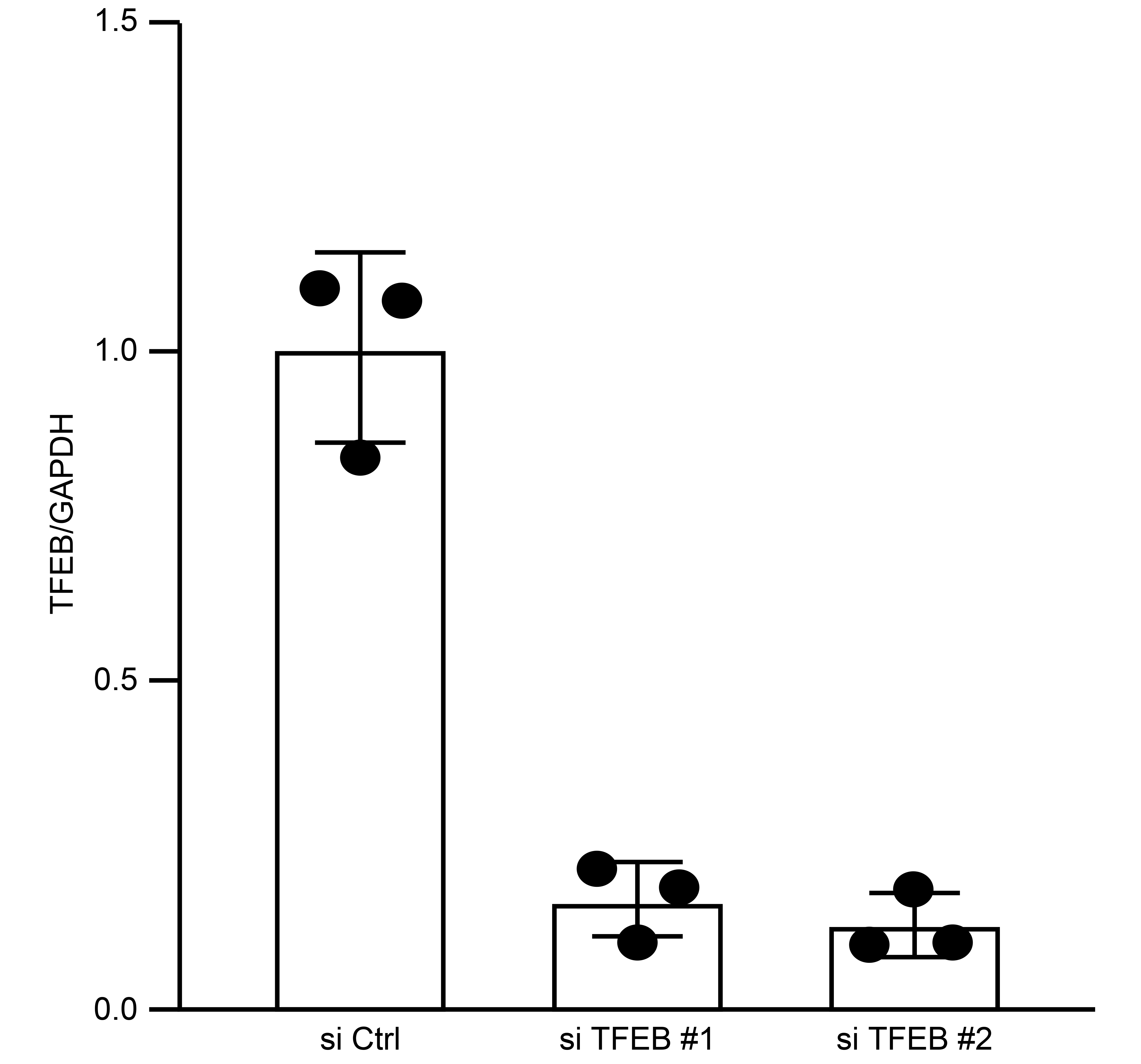

Supplement: Supplementary file 5 — Figure S5 [file 41420_2024_2139_MOESM5_ESM.tif]

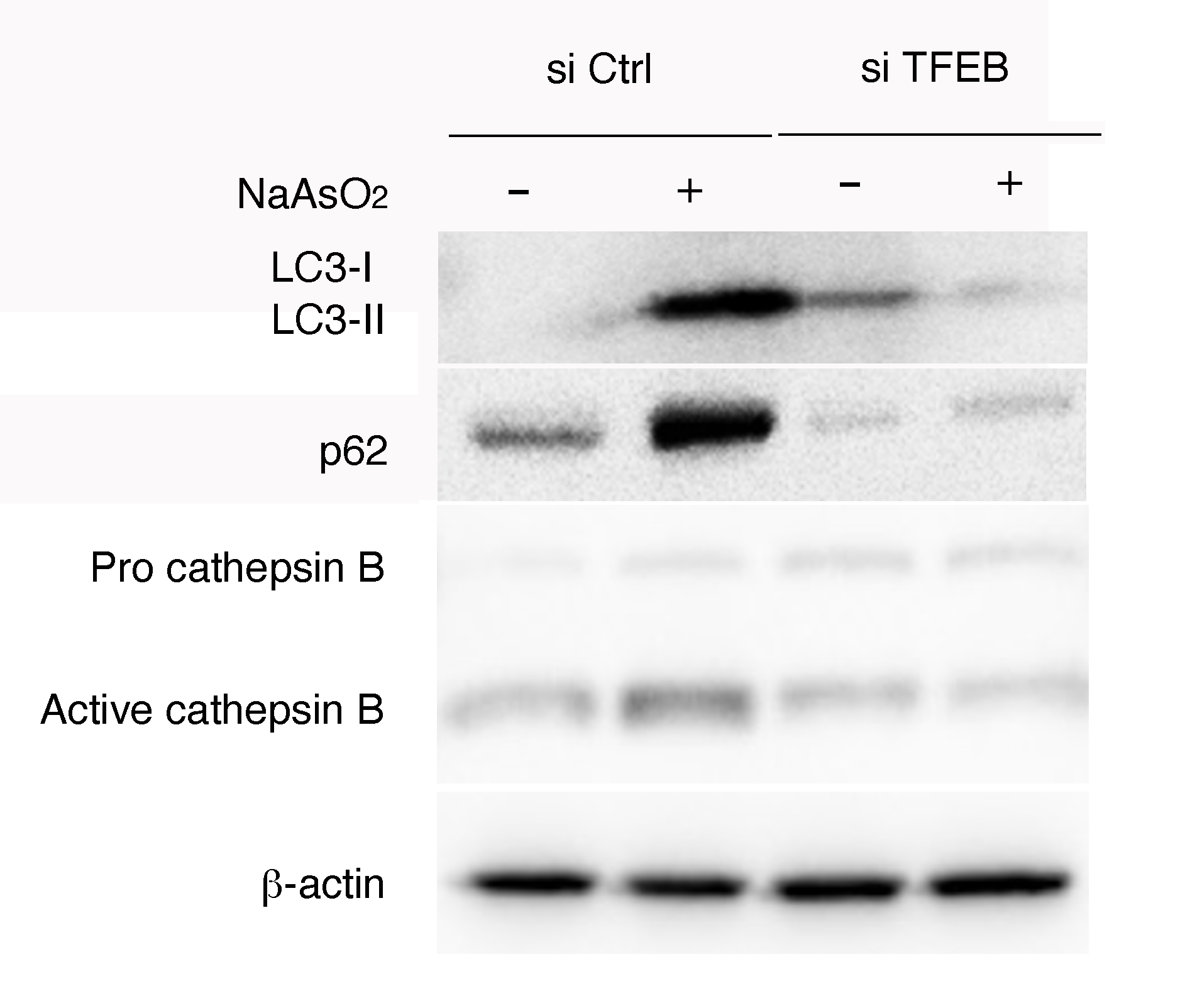

Supplement: Supplementary file 6 — Figure S6 [file 41420_2024_2139_MOESM6_ESM.tif]

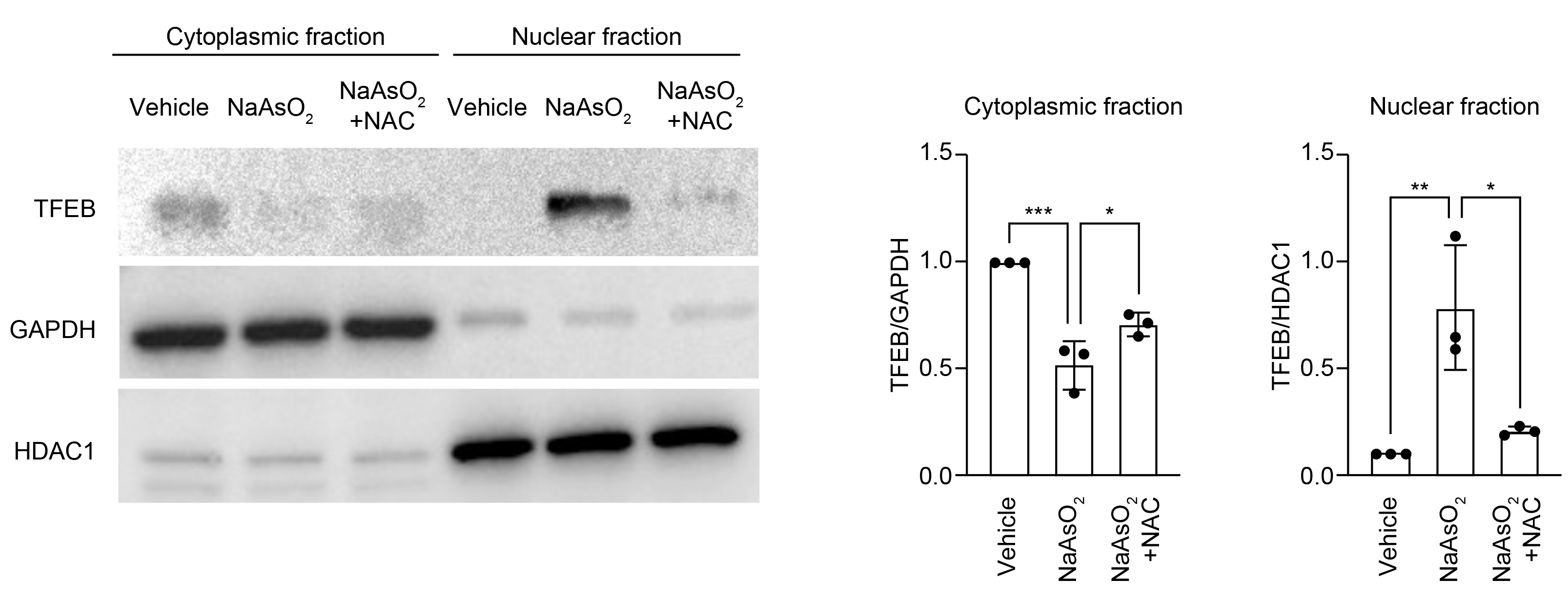

Supplement: Supplementary file 7 — Figure S7 [file 41420_2024_2139_MOESM7_ESM.tif]
