# Supplementary material for: Premature senescence is regulated by crosstalk among TFEB, the autophagy lysosomal pathway and ROS derived from damaged mitochondria in NaAsO2-exposed auditory cells
Source: Cell Death Discov. 2024 Aug 28;10:382. doi: 10.1038/s41420-024-02139-4 (PMC11350138; doi:10.1038/s41420-024-02139-4)
Supplement: Supplementary file 8 — Supplementary Table S1-3 [file 41420_2024_2139_MOESM8_ESM.docx]

Table S1. The sequencing primers for qPCR

| Gene | Forward primer (5’-3’) | Reverse primer (5’-3’) |
| --- | --- | --- |
| *Cdkn2a* (p16) | GAACTCTTTCGGTTCGTACCC | CGAATCTGCACCGTAGTTGA |
| *Cdkn1a* (p21) | GGAACATCTCAGGGCCGAAA | TCCTGACCCACAGCAGAAGA |
| *Il6* | GTCGGAGGCTTAATTACACA | TCTGAAGGACTCTGGCTTTG |
| *Il1b* | CAGGATGAGGACATGAGCACC | CTCTGCAGACTCAAACTCCAC |
| *Il8* | GTCCTTAACCTAGGCATCTTCG | TCTGTTGCAGTAAATGGTCTCG |
| *Cxcl10* | GCCGTCATTTTCTGCCTCA | CGTCCTTGCGAGAGGGATC |
| *Map1lc3b* (LC3B) | CGTCCTGGACAAGACCAAGT | ATTGCTGTCCCGAATGTCTC |
| *Sqstm1* (p62) | GCTGAAGGAAGCTGCCCTAT | TTGGTCTGTAGGAGCCTGGT |
| *Lamp1* | CAGCACTCTTTGAGGTGAAAAAC | CCATTCGCAGTCTCGTAGGTG |
| *Ctsb* (CathepsinB) | GAAGAAGCTGTGTGGCACTG | GTTCGGTCAGAAATGGCTTC |
| *Ctsd* (CathepsinD) | AGGTGAAGGAGCTGCAGAAG | ATTCCCATGAAGCCACTCAG |
| *Tfeb* | AAGGTTCGGGAGTATCTGTCTG | GGGTTGGAGCTGATATGTAGCA |
| *Gapdh* | TGCACCACCAACTGCTTAG | GATGCAGGGATGATGTTC |

Table S2. The dilutions of individual primary antibodies for western blot

| Primary antibodies | Dilution rate |
| --- | --- |
| Mouse anti-p21 antibody (Santa Cruz, sc-6246) | 1:200 |
| Mouse anti-16 antibody (Santa Cruz, sc-377412) | 1:200 |
| Rabbit anti-γ-H2AX (Ser139) antibody (Cell Signaling Technology, #9718) | 1:1000 |
| Rabbit anti-LC3 antibody (MBL, PM036) | 1:1000 |
| Rabbit anti-SQSTM1/p62 antibody (Cell Signaling Technology, #5114) | 1:1000 |
| Rabbit anti-Cathepsin B antibody (Cell Signaling Technology, #31718) | 1:1000 |
| Rabbit anti-TFEB antibody (Cell Signaling Technology, #32361) | 1:1000 |
| Mouse anti-TFE3 antibody (Proteintech, 67319-1-lg) | 1:2000 |
| Mouse anti-HDAC１antibody (Cell Signaling Technology, #5356) | 1:1000 |
| Rabbit anti-GAPDH antibody (Cell Signaling Technology, #2118) | 1:1000 |
| Mouse anti-β-actin antibody (Dako, A5316) | 1:10000 |

Table S3. The dilutions of individual secondary antibodies for western blot

| Secondary antibodies | Dilution rate |
| --- | --- |
| HRP-linked anti-mouse antibodies (Cell Signaling Technology, #7076) | 1:3000 |
| HRP-linked anti-rabbit antibodies (Cell Signaling Technology, #7074) | 1:3000 |
| Anti-rabbit IgG antibody (Alexa Fluor^®^ 555 Conjugate) (Cell Signaling Technology, #4413) | 1:3000 |
