# Supplementary material for: Premature senescence is regulated by crosstalk among TFEB, the autophagy lysosomal pathway and ROS derived from damaged mitochondria in NaAsO2-exposed auditory cells
Source: Cell Death Discov. 2024 Aug 28;10:382. doi: 10.1038/s41420-024-02139-4 (PMC11350138; doi:10.1038/s41420-024-02139-4)
Supplement: Supplementary file 9 — Supplementary figure legends [file 41420_2024_2139_MOESM9_ESM.docx]

**Supplementary figure legends**

**Fig. S1. Original full-length western blots.**

(A) Fig. S2B, (B) Fig. 2B, (C) Fig. 3A (left panel and right panel), (D) Fig. S3A, (E) Fig. 4A, (F) Fig. S4, (G)Fig. S6, (H) Fig. S7.

**Fig. S2. A premature senescence phenotype in HEI-OC1 cells subjected to short-term NaAsO_2_ exposure.**

(A) Representative immunofluorescence image (left) and quantification of γH2AX foci per cell (right) 1 day after short NaAsO_2_ exposure. The number of γH2AX foci per cell was counted under an inverted fluorescence phase-contrast microscope (BioZero BZ-8100 All-In-One Fluorescence Microscope, Keyence, Osaka, Japan) and quantified with ImageJ (NIH). DAPI was used to counterstain DNA in the nucleus for cell identification. Cells were quantified by counting more than 100 cells for each sample. All values are the means ±S.D.s from three or more independent experiments. ***p<0.001, Student's *t* test. Scale bar, 50 μm.

(B) Left panel: Relative mRNA expression of the p16 and p21 genes was measured via RT‒qPCR 1 day after short NaAsO_2_ exposure. Right panel: Representative western blots showing p16 and p21 expression in cells subjected to a short exposure to NaAsO_2_ at the indicated time points. β-Actin was used as a loading control. All values are the means ±S.D.s from three or more independent experiments. **p<0.01, ns: not significant, Student's *t* test. Full-length blots are presented in Supplementary information Fig. S1A.

(C) Left panel: The cell morphology after short-term NaAsO_2_ exposure (250 μM for 1 h) was observed under a light microscope. Cells indicated with arrows (→) show the typical characteristics of senescent cells (an enlarged, flattened and irregular morphology). Scale bar: 100 μm. Right panel: The mean area of SA-βgal-positive cells. The area was measured with ImageJ (NIH). All values are the means ±S.D.s from three or more independent experiments. *p<0.05, ***p<0.001, ns: not significant, Student's *t* test.

**Fig. S3. NaAsO_2_-induced premature senescence in HEI-OC1 cells is deeply involved to the degradation capacity of the autophagy‒lysosome pathway.**

(A) Autophagic flux assay. HEI-OC1 cells exposed to NaAsO_2_ (500 μM for 1 h) were incubated with bafilomycin A1 (100 nM for 24 h) and subjected to western blot analysis. β-Actin was used as a loading control. The expression of LC3-II was evaluated as an indicator of autophagic flux. All values are the means ±S.D.s from three or more independent experiments. *p<0.05, **p<0.01, ***p<0.001, ns: not significant, Tukey’s test. Full-length blots are presented in Supplementary information Fig. S1D.

(B) HEI-OC1 cells exposed to NaAsO_2_ (500 μM for 1 h) were incubated with bafilomycin A1 (100 nM for 24 h) or chloroquine (50 μM for 24 h) and subjected to SA-βgal staining. SA-βgal-positive cells were quantified by counting more than 200 cells for each sample 3 days after this treatment, and the positive rate was calculated from the ratio of positive cells to total cells. The control condition exhibited no detectable SA-βgal staining. All values are the means ±S.D.s from three or more independent experiments. **p<0.01, ****p<0.0001, Tukey’s test.

(C) HEI-OC1 cells exposed to NaAsO_2_ (500 μM for 1 h) were incubated with rapamycin (10 nM for 1 h) and subjected to SA-β-gal staining. SA-β-gal-positive cells were quantified by counting more than 200 cells in each sample 3 days after treatment, and the positive rate was calculated from the ratio of positive cells to total cells. The control condition exhibited no detectable SA-β-gal staining. All values are the means ±S.D.s from three or more independent experiments. **p<0.01, ****p<0.0001, Tukey’s test.

(D) Representative transmission electron microscopy (TEM) photomicrographs of cells from the vehicle group, cell at 24 h after short-term exposure to NaAsO_2_ (500 μM for 1 h) and EBSS-starved cells. (1) The structure of the nucleus in the vehicle group was normal, and the cytoplasm also had normal-appearing lysosomes, mitochondria and endoplasmic reticulum (10,000×). Scale bar: 500 nm. (2) Healthy mitochondria and endoplasmic reticulum were observed in the vehicle group. (3) Both damaged and healthy mitochondria were observed in cells subjected to short-term exposure to NaAsO_2_ (500 μM for 1 h) (10,000×). Scale bar: 500 nm. (4) Damaged mitochondria exhibited morphological characteristics consisting of abnormal swelling, crista collapse and stacks of crista membranes. (5) The immature form of autophagosomes, autolysosomes and multivesicular bodies (MVBs) or aggregates present in cells subjected to short-term exposure to NaAsO_2_ (500 μM for 1 h) (10,000×). Scale bar: 200 nm. (6) The degradation of dense organelles into immature autophagosomes, autolysosomes and MVBs remained incomplete. (7) Healthy autophagosomes, autolysosomes, MVBs and endosomes present in EBSS-starved cells (10,000×). Scale bar: 200 nm. (8) Autolysosomes have a perfectly round shape with double membranes. Degradation was completed in autolysosomes.

**Fig. S4. The expression of TFEB in the nuclear and cytoplasmic fractions of HEI-OC1 cells subjected to a short exposure to NaAsO_2._**

The expression of TFEB was increased in a dose-dependent manner in the nuclear fraction of HEI-OC1 cells after a short exposure to NaAsO_2_, and it was decreased in a dose-dependent manner in the cytoplasmic fraction, revealing a clear inverse correlation. The peak expression of TFEB at 6 h in EBSS-starved cells was used as a positive control.

**Fig. S5. TFEB knockdown using two different siRNAs in HEI-OC1 cells.**

TFEB expression was knocked down with two different short interfering RNAs (siRNAs; #1: Santa Cruz Biotechnology, Inc., CA, USA and #2: Dharmacon Technologies, Lafayette, CO, USA).

**Fig. S6. The expression of autophagy-related proteins (LC3-Ⅱ and p62) and lysosomal proteins (pro- and active cathepsin B) was not induced in TFEB-KD HEI-OC1 cells after short-term exposure to NaAsO_2_.**

In HEI-OC1 cells transfected with TFEB siRNA (Dharmacon Technologies, Lafayette, CO, USA) or a nontargeting siRNA (si-Ctrl), the expression of LC3-Ⅱ, p62 and pro- and active-cathepsin B at 6 h after no exposure (negative control) or short-term exposure to NaAsO_2_ (500 μM for 1 h) was assessed by western blot analysis. Western blot analysis was performed as described in the Materials and Methods section. All values are the mean ± S.D. from three or more independent experiments. *p< 0.001, **p< 0.0001. The full blots are presented in Supplementary information **Fig. S1G.**

**Fig. S7. The effect of NAC on the expression of TFEB in the nuclear fraction of HEI-OC1 cells.**

NAC completely decreased the expression of TFEB in the nuclear fraction but increased TFEB in the cytoplasmic fraction. The expression of TFEB at the protein level was measured by western blots at 12 h after treatment with NAC (2 mM for 1 h) and NaAsO_2_ (500 μM for 1 h) in the same way. Western blot analysis was performed as described in the Materials and Methods. β-Actin was used as a loading control. All values are the means ±S.D.s from three or more independent experiments. AS: NaAsO_2_. *p<0.05, **p<0.01, ***p<0.001, ****p<0.0001, ns: not significant, Tukey’s test. Full-length blots are presented in Supplementary information Fig. S1H.
